# Associations between diet quality indices and psoriasis severity: results from the Asking People with Psoriasis about Lifestyle and Eating (APPLE) cross-sectional study

**DOI:** 10.1017/S0007114525000340

**Published:** 2025-02-28

**Authors:** Sylvia Zanesco, Thiviyani Maruthappu, Christopher E. M. Griffiths, Kathryn V. Dalrymple, Rachel Gibson, Wendy L. Hall

**Affiliations:** 1 Department of Nutritional Sciences, School of Life Course & Population Sciences, Faculty of Life Sciences & Medicine, King’s College London, 150 Stamford Street, London SE1 9NH, UK; 2 St John’s Institute of Dermatology, School of Basic and Medical Biosciences, King’s College London, 2 Lambeth Palace Road, London SE1 7EP, UK

**Keywords:** Diet, Nutrition, Psoriasis, Mediterranean diet, Dietary Approaches to Stop Hypertension, diet, Skin

## Abstract

Psoriasis is a chronic immune-inflammatory skin disease. Cross-sectional research examining diet quality indices in psoriasis has focused on the Mediterranean diet and is confined to Mediterranean populations, thereby lacking generalisability to other populations. We explored associations between diet quality indices and the likelihood of reporting a higher psoriasis severity. This was an online cross-sectional study recruiting adult volunteers with psoriasis (*n* 257). A 147-item FFQ evaluated adherence to the Mediterranean diet score, the Dietary Approaches to Stop Hypertension score and the Plant-based Diet Index (PDI), including its original (oPDI), healthy (hPDI) and unhealthy (uPDI) subtypes. Psoriasis severity was determined with the self-assessed Simplified Psoriasis Index. When adjusted for age, sex, smoking, alcohol overconsumption, energy intake and psychological morbidity, multinomial logistic regression analyses revealed an increased likelihood of reporting a higher psoriasis severity in participants with a very low adherence to Dietary Approaches to Stop Hypertension (OR = 3·75; 95 % CI 1·313, 10·700; *P* = 0·01) and hPDI (OR = 4·04; 95 % CI 1·251, 13·064; *P* = 0·02) patterns. A reduced likelihood of reporting a higher psoriasis severity was shown in participants with low adherence to the uPDI (OR = 0·25; 95 % CI 0·085, 0·716; *P* = 0·01). With further adjustment for BMI, a very low adherence to the oPDI was significantly associated with an increased likelihood of reporting a higher psoriasis severity (OR = 3·46; 95 % CI 1·029, 11·656; *P* = 0·05). Dietary interventions and assessment should be introduced in the care pathway for psoriasis management.

Diet is increasingly recognised as a therapeutic tool to prevent and manage chronic diseases and has been implicated in the pathogenesis of inflammatory conditions^(1,2)^. Psoriasis is a chronic skin disease presenting as red, heavily scaled plaques, most commonly on the extensor elbows and knees, lower back and scalp, which significantly impairs life quality^(3,4)^. Interactions between genetic predisposition and environmental factors are key to the manifestation of psoriasis^(5)^. Partly because of the underlying systemic inflammation, people with psoriasis are at an increased risk of developing cardiometabolic morbidities^(6)^.

Psoriasis has a multifactorial aetiology, including modifiable triggers such as smoking, alcohol and obesity, with relapsing and remitting symptoms. The contributing role of diet to the chronic course of psoriasis is unclear, and robust evidence is lacking^(7)^. Poor adherence to the American Heart Association guidelines was associated with a 43 % increased risk of incident psoriasis in the UK Biobank study^(8)^, an effect which was amplified when compounded with smoking, adiposity and physical inactivity. The Copenhagen General Population Study showed that non-adherence to national healthy eating guidelines was associated with an increased risk for prevalent psoriasis, although confounder adjustments attenuated associations^(9)^.

According to the results of the NutriNet Santé cohort, the risk of severe psoriasis was inversely associated with adherence to the Mediterranean diet (MD) as assessed by the MEDI-LITE score^(10)^. Inverse associations between psoriasis severity and MD adherence have repeatedly emerged using diet quality indices (DQI) such as the PREvención con DIeta MEDiterránea (PREDIMED) and the MedDietScore^(11–13)^. An important limitation is that these findings are limited to Mediterranean populations. To our knowledge, no study has tested associations between DQI such as the Dietary Approaches to Stop Hypertension (DASH), recognised as a healthy eating pattern originating in North America^(14)^, or the Plant-based Diet Indices (PDI)^(15)^, examining the proximity to increasingly popular pro-vegetarian dietary trends, and psoriasis severity in a UK-based population.

To align with the first research priority of the psoriasis priority settings partnership^(16)^, we evaluated associations between adherence to DQI and the likelihood of reporting a higher psoriasis severity in UK-based adults with psoriasis. A secondary aim was to investigate associations between individual DQI components and psoriasis severity.

## Methods

### Design

The Asking People with Psoriasis about Lifestyle and Eating (APPLE) study (NCT05448352) was a cross-sectional observational study delivered as an open online survey (https://osf.io/cdbgh/files/osfstorage/66317c6c4664da0185ed6ae5). This survey collected information on diet, lifestyle and psoriasis severity of people living with psoriasis in the UK. This manuscript was written according to the Strengthening the Reporting of Observational Studies in Epidemiology – Nutrition (STROBE-NUT)^(17)^ and the Checklist for Reporting Results of Internet E Surveys (STROBE – CHERRIES)^(18)^(online Supplementary Information 1 and 2). The study was approved by the King’s College London Research Ethics Committee (LRS/DP-21/22-29257) and the London – Westminster National Health Service Research Ethics Committee (23/LO/0536).

### Survey development

The survey was developed on Qualtrics XM (Qualtrics International Inc – https://www.qualtrics.com/uk/). Before fielding the survey, the usability and technical functionality were piloted by steering group members, dermatologists and lay people with psoriasis (*n* 8). Amendments to the initial survey were made according to the feedback provided on language, questions and survey logic. The final survey comprised of 131 items, unevenly distributed across 14 sections with a completeness check present at the item level under a response validation for each item.

### Study population

Eligible participants were adults (18+ years) living with psoriasis, residing in the UK and proficient in the English language. Participants self-reported their eligibility. Participation in the study was voluntary, and participants could terminate the study at any time up to the point of submission. As an incentive, participants were invited to attend a ‘Nutrition in Psoriasis’ webinar.

### Data handling

Volunteers were required to click to confirm to have read the information sheet to proceed with electronic informed consent (e-consent). To e-consent, volunteers were required to click on each informed consent statement. The built-in survey logic would not let volunteers access the APPLE study survey without e-consent. Participants were able to review answers by pressing the back button. Once the survey was submitted, answers could not be amended. Participants could withdraw their data from the study upon request.

Identifiable information (name and email address) was only accessible to the nutritionist (SZ). Survey entries were pseudonymised and assigned a unique identifier using a pseudonym code break spreadsheet. The survey entries and pseudonym code break spreadsheet were password protected and stored on a SharePoint drive, accessible only by SZ and the principal investigator (WLH).

Cookies were used to save the survey responses, which were valid for 7 d. If a participant closed the survey, the survey could be resumed at a later date, where it was left off, by clicking on the survey link on the same browser and on the same device (provided cookie data were not deleted). After 7 d, the survey responses were recorded on Qualtrics as incomplete. Upon study completion, IP addresses were scanned to identify duplicate entries from the same participant. Duplicate entries with the same IP address were eliminated before analysis. The initial most complete response from a duplicate entry was retained for analysis.

Incomplete survey responses (less than 50 % completion) were excluded. Incomplete survey responses (with more than 50 % completion) were included in the analysis where data were available. For the computation of diet scores, only participants with complete survey responses were included. Outputs with missing data are denoted in the footnotes.

### Recruitment

Participants were recruited by convenience sampling between the 18th of June 2022 and the 8th of January 2024. The survey was accessible on a landing page https://dietandpsoriasisproject-apple.com, which contained a ‘Meet the Team’, ‘Contact us’ and ‘Frequently Asked Questions’ section. No initial contact was made with potential participants. The study was advertised on social media (online Supplementary Information 3) and shared internally via the King’s College London recruitment newsletter. Gatekeeper approval was obtained by the Psoriasis Association (PA). The PA assisted with recruitment by circulating an email to the PA research network, a member community actively engaged in research, and by posting adverts on the PA’s social media platforms, newsletters and magazines.

### Assessment of diet quality

Dietary information was collected with a validated and modified European Prospective Investigation into Cancer FFQ (EPIC FFQ)^(19)^. Modifications to the original FFQ include the introduction of 20 food items and the omission of four food items, for a total of 147 food items (online Supplementary Information 4). Dietary data were converted into average daily quantities of food items by multiplying the frequency of consumption per the standard portion size relative to that food item. Energy and nutrient data were calculated per the Composition of Food Integrated Dataset^(20)^. Participants with dietary intakes of < 500 or > 3500 kcal/d for women and < 800 or > 4200 kcal for men were omitted from the analysis^(21)^.

### Assessment of psoriasis severity

Psoriasis severity was self-assessed with the self-assessed Simplified Psoriasis Index (sa-SPI). This is a validated self-reported measure, generating a score between 0 and 70 points based on three components: severity, psychosocial impact and intervention history^(22)^. The *severity* component uses a 3-point scale to rate the severity of psoriasis on ten body parts in response to the question ‘which best describes your psoriasis today?’: ‘clear or so minor that it does not bother me’ (0 points), ‘obvious but still leaving plenty of normal skin’ (0·5 points) and ‘widespread and involving much of the affected area’ (1 point). An overall rating of the skin is included, with ‘clear’ (0 points) ranging to ‘intensely inflamed skin’ (5 points). A 10-point scale ranging from 0 = not at all (0 points) to 10 = very much (10 points) evaluated the *psychosocial* component. For the *intervention history* component, participants indicated which of the four statements applied to them, for example, ‘I have had psoriasis for at least 10 years’, scoring 1 point per statement selected, and required the participant to select the psoriasis treatments received (e.g. methotrexate) as part of their care plan, scoring 1 point per treatment selected, for a maximum of 6 points. Scores between 0 and 9 points indicated mild psoriasis, 10–19 points indicated moderate psoriasis and 20–70 points indicated severe psoriasis.

Responses to the sa-SPI correlate with the Psoriasis Area Severity Index, the gold-standard measure for clinically assessing psoriasis severity^(23)^ and to the Dermatology Life Quality Index, a self-report questionnaire evaluating the day-to-day impact of dermatoses^(24)^, illustrating its validity and reliability in providing a comprehensive outlook on psoriasis severity that is not just limited to clinical presentations^(22,25)^.

### Assessment of covariates

Participants self-reported their age, sex, weight, height and smoking status. Diagnoses of depression and anxiety were self-disclosed. If participants responded ‘yes’ to ‘Depression’ or ‘Anxiety’ in relation to the question ‘Have you ever been medically diagnosed with any of the following conditions?’ the participant was considered to have a psychological morbidity, which was evaluated as a dichotomous covariate. Alcohol overconsumption was assessed as a continuous variable using the Alcohol Use Disorders Identification Test Consumption scoring the frequency, units and overconsumption of alcohol using three 5-point Likert scale questions with a maximum of 12 points^(26)^. Weight and height were used to calculate BMI.

### *A priori* diet quality indices

The Mediterranean diet score (MDS) was selected because the MD is recognised as one of the healthiest diets in the world, with the original index having been adapted to measure food intakes of non-Mediterranean populations^(27)^. The DASH index was selected because it is representative of universal healthy eating guidelines. The PDI represents a pro-vegetarian style dietary pattern focusing on healthier, less pro-inflammatory plant-based foods^(28)^ and was selected to align with emerging dietary trends. The FFQ components contributing to the DQI are shown in online Supplementary Information 5.

### The Mediterranean diet score

Adherence to the MD was measured with the standard MDS^(29)^. The MDS is derived from a nine-component protocol assigning points based on sex-specific medians. Vegetables, fruits and nuts, wholegrains, legumes and fish were positively scored: 0 points if intakes are lower than the median and 1 point if above the median. Dairy products and meat and poultry were negatively scored: 1 point if lower than the median and 0 points if above the median. The monounsaturated-to-saturated fat ratio was positively scored: 1 point with a ratio equal or above 1 and 0 points with a ratio less than 1. The original MDS included alcohol consumption per the Greek sex-specific alcohol recommendations. For this study, the MDS was adapted to the UK guideline of no more than 14 units per week^(30)^, which equates to 112 g/week or 16 g/d, as the cut-off value to score alcohol: 0 points if above 16 g/d and 1 point if below 16 g/d. The total MDS ranged from 0 to 9 points; higher scores represent higher adherence.

### Dietary Approaches to Stop Hypertension score

Adherence to the DASH pattern was determined using an eight-component protocol with rankings based on sex-specific quintiles^(14)^. For each participant, beneficial components (fruits, vegetables, nuts and legumes, wholegrains and low-fat dairy products) were assigned 1 point for the lowest quintile and up to 5 points for the highest quintile. Detrimental components (sodium, red and processed meat and sugar-sweetened beverages) were reverse scored and assigned 5 points for the lowest quintile and 1 point for the highest quintile. The total DASH score ranged between 8 and 40 points; higher scores represent higher adherence.

### Plant-based Diet Index

Three PDI scores, including the original PDI (oPDI), healthy PDI (hPDI) and unhealthy PDI (uPDI) subtypes, were obtained from seventeen food components^(15)^. For this study, the original eighteen-component PDI was modified to omit the vegetable oil component, composed of vegetable oils used for cooking and oil-based salad dressing intakes, which are not captured in the modified EPIC FFQ. The PDI ranks quintiles of intakes across three main groups of food and beverage components: healthy plant foods, less healthy plant foods and animal foods. For all PDI subtypes, components in the animal food group were negatively scored: 5 points for the lowest quintile and 1 point for the highest quintile. The oPDI positively scores components in both the healthy and less healthy plant food groups, allocating 1 point for the lowest quintile and 5 points for the highest quintile. In contrast, the hPDI positively scores only the components in the healthy plant food group and negatively scores components in the less healthy plant food group. The uPDI negatively scores components in the healthy plant food group and positively scores the components of the less healthy plant food groups. The PDI ranges from 17 to 85 points, with higher scores representing higher adherence to the respective PDI.

### Statistical analysis

The number of unique site visitors was determined as the total number of unique IP addresses that accessed the survey. The view rate was calculated by dividing the number of respondents who clicked to have read the information sheet by the total unique site visitors. The participation rate was calculated by dividing the number of respondents who provided informed consent by the number of respondents who clicked to have read the information sheet. The completion rate was determined by dividing the number of respondents who completed the survey by the number of respondents who provided informed consent.

IBM SPSS Statistics version 29.0.0.0 was used for the analysis. Distributions were determined by visually inspecting histograms and Q-Q plots. Baseline demographic, anthropometric and lifestyle data were reported in descriptive statistics, including the median (interquartile range) for continuous variables and frequency (%) for categorical variables. To test linear associations, the *a priori* DQI and sa-SPI scores were transformed by fractional ranking using the inverse distribution function for normality. Correlations between DQI and psoriasis severity were analysed with Pearson’s correlation coefficient. Multinomial logistic regression analyses determined the OR and 95 % CI of severe psoriasis associated with DQI adherence. To do this, the normalised DQI and sa-SPI scores were rank transformed into ordinal variables. The MDS and sa-SPI were rank transformed into tertiles as the MDS is out of 9 points and psoriasis severity is usually classified into three groups. The DASH and PDI were rank transformed into quintiles as the scores have a wider range (from 17 to 85 points). For interpretation purposes, quintiles for adherence to DASH and PDI patterns (Q_1_, Q_2_, Q_3_, Q_4_ and Q_5_) were classified as ‘very low adherence’, ‘low adherence,’ ‘modest adherence’, ‘high adherence’ and ‘very high adherence’. Tertiles for MDS adherence (T_1_, T_2_ and T_3_) were categorised as ‘low adherence’, ‘modest adherence’ and ‘high adherence’. For psoriasis severity, the sa-SPI tertiles (T_1_, T_2_ and T_3_) were interpreted as ‘low psoriasis severity’, ‘increasing psoriasis severity’ and ‘high psoriasis severity’. The mean (SD) for MDS and sa-SPI tertiles and DASH and PDI quintiles are tabulated in online Supplementary Information 6. Confounder adjustments were executed in a sequence of additive models adjusting for demographic characteristics (model I) and building on knowledge of known associated covariates (models II–V). Adjustments were as follows: model I: age (years, continuous), sex (male or female), smoking status (active smoker or non-smoker); model II: model I and Alcohol Use Disorders Identification Test Consumption score (continuous); model III: model II and energy intake (continuous in kcal/d); model IV: model III and psychological morbidity (yes or no); model V: model IV and BMI (continuous). As a secondary analysis, we conducted a stepwise multiple linear regression as described by Barrea and colleagues to estimate the predictive effect of individual score components on psoriasis severity; components with a variance inflation factor above 10 were excluded to avoid multicollinearity^(11)^. Psoriasis severity was the dependent variable, and the individual score components were the independent variables, added in a stepwise addition to the model. Statistically significant components (*P* < 0·05) were further tested with univariate regression analyses adjusting for models I–V. A mediation analysis was conducted to clarify whether BMI mediated the DQI–psoriasis severity relationships.

## Results

### Response rates

**Figure 1.**
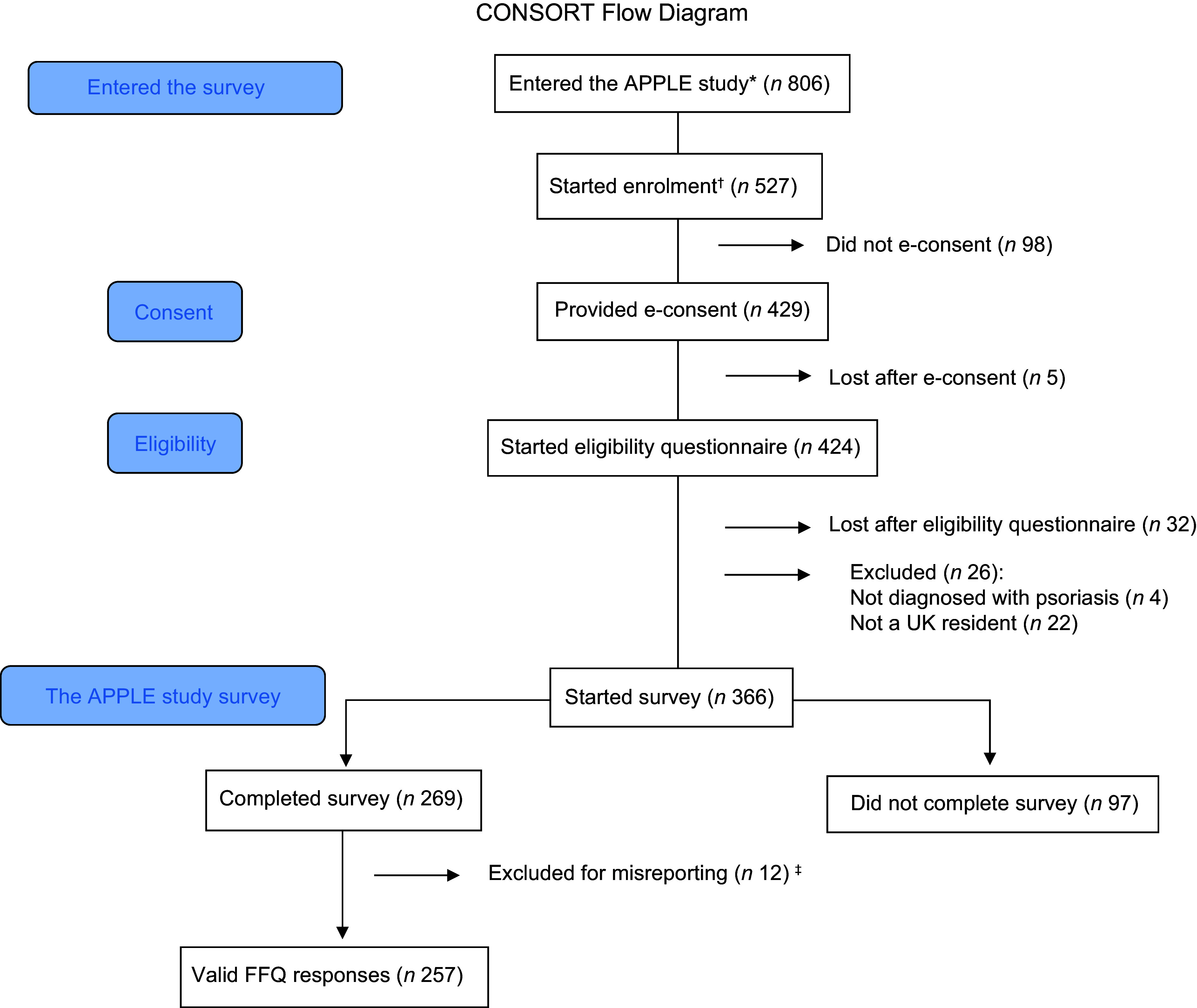
CONSORT flow diagram. APPLE, Asking People with Psoriasis about Lifestyle and Eating. *number of unique IP addresses that accessed the survey. †number of volunteers who confirmed to have read the information sheet. ‡*n* 1 and *n* 11 excluded for underreporting and overreporting, respectively.

The flow of participants in the APPLE study is shown in Figure 1. There was a total of 806 unique site visitors, of which 429 provided informed consent. This translates into a view rate of 65 % and a participation rate of 81 %. Three-hundred and sixty-six volunteers started the APPLE study survey, of which 27 % (*n* 97) had partially completed survey responses with missing anthropometric, dietary or psoriasis severity data. The remaining 269 (73 %) participants had complete survey responses (except for 3 participants with missing weight measures). A further twelve participants (4 %) were omitted from the DQI computation for misreporting energy intakes^(21)^. The final sample size was 257 participants, yielding a completion rate of 60 %. Two-thirds of participants with incomplete responses reported to have overweight or obesity, whilst 51·5 % of participants with complete responses reported to have a BMI ≥ 25·0 kg/m^2^. A lower proportion of participants with incomplete responses reported a psychological morbidity (26·2 %), compared with participants with complete survey responses (45·0 %) (online Supplementary Information 7).

### Demographics

Table 1 describes the demographic characteristics of the 257 participants with valid FFQ responses. The sample population was predominantly female (82·5 %), of white-British ethnicity (84·8 %), with a median age of 40 years (lower and upper limits of the interquartile range (IQR) 31–51 years) and a median BMI of 25 kg/m^2^ (IQR 22–30 kg/m^2^). Respondents were mainly non-smokers (82·1 %) with a low risk of alcohol overconsumption (59·9 %). Psoriasis severity was reported as mild (24·1 %), moderate (44·7 %) and severe (31·2 %). Half of the study population reported a family history of psoriasis (53·3 %). Psoriasis-related comorbidities were reported as follows: psoriatic arthritis (22·2 %), cardiometabolic disorders (19·5 %), psychological conditions (44·7 %) and gastrointestinal diseases (21·0 %).

**Table 1.** Demographic characteristics of the APPLE study participants with valid FFQ responses (*n* 257) (Numbers and percentages; median values and interquartile ranges)

	*n*	%
Age, years		
Median	40	
IQR	20	
Sex (*n*, %)		
Male	45	17·5
Female	212	82·5
BMI		
Median	25	
IQR	8	
BMI classification (*n*, %)		
Underweight	7	2·8
Normal weight	118	46·5
Overweight	67	26·4
Obesity	62	24·3
Ethnicity (*n*, %)		
White – British	218	84·8
White (Other)	16	6·3
Mixed	10	3·8
South Asian	9	3·5
Asian (Other)	3	1·2
East Asian	1	0·4
Smoking status (*n*, %)		
Non-smoking	211	82·1
Actively smoking	45	17·5
Preferred not to say	1	0·4
Alcohol overconsumption (*n*, %)		
Low risk of dependency	154	59·9
Increasing risk of dependency	80	31·1
Higher risk of dependency	20	7·8
Possible dependence	3	1·2
Family history of psoriasis (*n*, %)		
Yes	137	53·3
No	120	46·7
Morbidity (*n*, %)		
Psoriatic arthritis		
Yes	57	22·2
No	200	77·6
Cardiometabolic		
Yes	50	19·5
No	207	80·5
Psychological		
Yes	115	44·7
No	142	55·3
Gastrointestinal		
Yes	54	21·0
No	203	79·0
Psoriasis severity (*n*, %)		
Mild	62	24·1
Moderate	115	44·7
Severe	80	31·2

*n* 3 missing values for BMI for lack of completeness.

Underweight BMI ≤ 17·99 kg/m^2^; normal weight BMI > 18·00 kg/m^2^ and ≤ 24·99 kg/m^2^; overweight BMI > 25·00 kg/m^2^ and ≤ 29·99 kg/m^2^; obesity BMI > 30·00 kg/m^2^.

Low risk of dependency 0–4 points; increasing risk of dependency 5–7 points; higher risk of dependency 8–10 points; possible dependence 11–12 points.

Cardiometabolic morbidity includes one or more diagnoses of heart disease, liver disease, stroke, type II diabetes, high blood pressure, high cholesterol or metabolic syndrome.

Psychological morbidity includes a diagnosis of depression or anxiety.

Gastrointestinal morbidity includes a diagnosis of irritable bowel syndrome, inflammatory bowel disease or celiac disease.

Psoriasis severity determined using the self-assessed Simplified Psoriasis Index (sa-SPI) classified with the standard sa-SPI cut-off ranges: mild psoriasis = 0–9·99 points, moderate psoriasis = 10–19·99 points and severe psoriasis > 20·00 points.

### Diet quality indices

**Figure 2.**
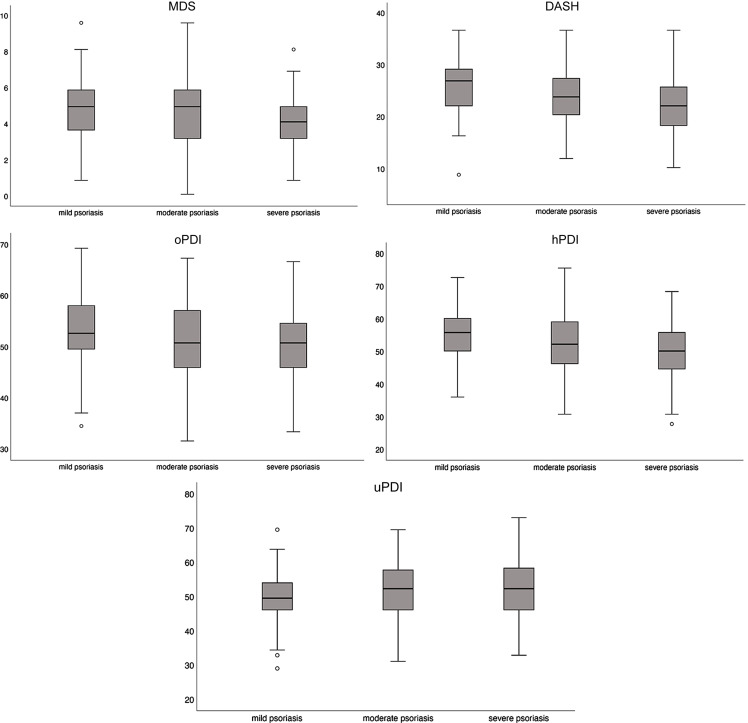
Box plots of the *a priori* DQI distributions according to psoriasis severity. DQI, diet quality index; MDS, Mediterranean diet score; DASH, Dietary Approaches to Stop Hypertension; oPDI, original Plant-based Diet Index; hPDI, healthy Plant-based Diet Index; uPDI, unhealthy Plant-based Diet Index.

The distributions of the DQI by psoriasis severity are shown in Figure 2. For the MDS (score range 0–9), the mean (SD) was 4·67 (1·66); for the DASH (score range 8–35), it was 23·88 (5·58), and for the PDI subtypes, the mean scores were 51·30 (7·29) for the oPDI, 52·29 (9·05) for the hPDI and 51·64 (8·34) for the uPDI (score range 17–85 for all).

Pearson correlation coefficients between the DQI and psoriasis severity are shown in Table 2. Only the uPDI reported no significant correlation with psoriasis severity. The remaining DQI were negatively correlated with psoriasis severity: DASH (*r* = −0·258, *P* < 0·001), hPDI (*r* = −0·203, *P* = 0·001), MDS *(r* = −0·183, *P* = 0·003) and oPDI (*r* = −0·175, *P* = 0·005).

**Table 2.** The mean (SD) of the DQI across psoriasis severity categories and Pearson’s correlation coefficients with psoriasis severity

	Psoriasis severity									
	Mild (*n* 62)		Moderate (*n* 115)		Severe (*n* 80)		Overall			
	Mean	sd	Mean	sd	Mean	sd	Mean	sd	*r*	*P*
MDS	4·85	1·68	4·82	1·68	4·36	1·60	4·67	1·66	–0·183	**0·003**
DASH	25·04	5·41	24·18	5·47	22·52	5·64	23·88	5·68	–0·258	**< 0·001**
oPDI	52·18	7·70	51·78	7·59	50·06	6·42	51·30	7·29	–0·175	**0·005**
hPDI	54·40	8·35	52·20	9·34	50·54	9·06	52·29	9·05	–0·203	**0·001**
uPDI	50·44	7·95	51·59	8·40	52·92	8·57	51·64	8·34	0·119	0·059

DQI, diet quality index; MDS, Mediterranean diet score; DASH, Dietary Approaches to Stop Hypertension; oPDI, original Plant-based Diet Index; hPDI, healthy Plant-based Diet Index; uPDI, unhealthy Plant-based Diet Index.

The means (SD) of the DQI are expressed as normalised values.

Psoriasis severity was determined using the self-assessed Simplified Psoriasis Index (sa-SPI).

sa-SPI: ≤ 10 points (mild psoriasis); 10–20 points (moderate psoriasis); 20–70 points (severe psoriasis).

MDS: ≤ 3 points (low adherence); 3–6 points (modest adherence); 6–9 points (high adherence).

DASH: ≤ 8 points (very low adherence); 8–16 points (low adherence); 16–24 points (modest adherence); 24–32 points (high adherence); 32–40 points (very high adherence).

oPDI: ≤ 17 points (very low adherence); 17–34 points (low adherence); 34–51 points (modest adherence); 51–68 points (high adherence); 68–75 points (very high adherence).

hPDI: ≤ 17 points (very low adherence); 17–34 points (low adherence); 34–51 points (modest adherence); 51–68 points (high adherence); 68–75 points (very high adherence).

uPDI: ≤ 17 points (very low adherence); 17–34 points (low adherence); 34–51 points (modest adherence); 51–68 points (high adherence); 68–75 points (very high adherence).

The bold face is for visual indication between significant and non-significant values.

Table 3 presents the unadjusted and adjusted (for models VI and V) multinomial regression analyses between quantiles of DQI and the OR (95 % CI) for psoriasis severity. Online Supplementary Information 8 reports confounder adjustments for models I–III. When adjusted for age, sex, smoking, Alcohol Use Disorders Identification Test Consumption, energy intake and psychological morbidity, very low adherence to the DASH (OR = 3·75; 95 % CI 1·313, 10·700; *P* = 0·01) and hPDI (OR = 4·04; 95 % CI 1·251, 13·064; *P* = 0·02) was associated with an increased likelihood of reporting higher psoriasis severity relative to very high adherence, which was also reported with a modest adherence to the MDS relative to high adherence (OR = 2·39; 95 % CI 1·102, 5·163; *P* = 0·03). A reduced likelihood of reporting high psoriasis severity was shown with low uPDI adherence relative to very high adherence (OR = 0·25; 95 % CI 0·085, 0·716; *P* = 0·01). When BMI was added as a covariate (model V), very low adherence to the oPDI was significantly associated with an increased likelihood of reporting a high psoriasis severity relative to very high adherence (OR = 3·46; 95 % CI 1·029, 11·656; *P* = 0·05), but a similar association for hPDI was no longer significant when adjusted for BMI.

**Table 3.** Diet quality indices and the unadjusted and adjusted OR (95 % CI) for psoriasis severity

			Unadjusted			Model IV			Model V		
		Cases/*n*	OR	95 % CI	*P*	OR	95 % CI	*P*	OR	95 % CI	*P*
MDS tertiles	Increased severity (T_2_) *v*. low severity (T_1_)										
	T_1_ low adherence	22/85	1·01	0·471, 2·158	0·983	1·01	0·453, 2·272	0·973	0·74	0·314, 1·721	0·478
	T_2_ modest adherence	33/85	1·40	0·684, 2·849	0·359	1·37	0·653, 2·867	0·406	1·18	0·555, 2·524	0·662
	T_3_ high adherence	30/85	Ref.								
	High severity (T_3_) *v*. low severity (T_1_)										
	T_1_ low adherence	28/88	1·83	0·847, 3·969	0·124	2·02	0·881, 4·623	0·097	1·14	0·462, 2·794	0·781
	T_2_ modest adherence	39/88	2·36	1·126, 4·934	**0·02**	2·39	1·102, 5·163	**0·03**	1·66	0·737, 3·739	0·221
	T_3_ high adherence	21/88	Ref.								
DASH quintiles	Increased severity (T_2_) *v*. low severity (T_1_)										
	Q_1_ very low adherence	15/85	2·03	0·734, 5·608	0·172	2·03	0·693, 5·919	0·197	1·27	0·406, 4·000	0·678
	Q_2_ low adherence	13/85	0·98	0·378, 2·526	0·962	0·93	0·353, 2·425	0·874	0·66	0·241, 1·811	0·420
	Q_3_ modest adherence	19/85	1·71	0·681, 4·312	0·253	1·63	0·632, 4·207	0·312	1·35	0·511, 3·562	0·545
	Q_4_ high adherence	21/85	1·67	0·683, 4·092	0·261	1·68	0·674, 4·164	0·267	1·51	0·602, 3·807	0·378
	Q_5_ very high adherence	17/85	Ref.								
	High severity (T_3_) *v*. low severity (T_1_)										
	Q_1_ very low adherence	24/88	3·45	1·301, 9·150	**0·01**	3·75	1·313, 10·700	**0·01**	1·70	0·538, 5·340	0·368
	Q_2_ low adherence	23/88	1·84	0·756, 4·461	0·179	1·81	0·726, 4·514	0·203	1·04	0·389, 2·784	0·936
	Q_3_ modest adherence	14/88	1·34	0·509, 3·533	0·552	1·33	0·487, 3·626	0·578	0·93	0·323, 2·644	0·884
	Q_4_ high adherence	11/88	0·93	0·345, 2·506	0·886	0·98	0·356, 2·684	0·965	0·77	0·269, 2·175	0·616
	Q_5_ very high adherence	16/88	Ref.								
oPDI quintiles	Increased severity (T_2_) *v*. low severity (T_1_)										
	Q_1_ very low adherence	17/85	1·13	0·438, 2·914	0·801	1·40	0·491, 3·971	0·531	1·14	0·393, 3·318	0·808
	Q_2_ low adherence	12/85	0·86	0·315, 2·368	0·776	0·94	0·331, 2·679	0·911	0·81	0·280, 2·352	0·700
	Q_3_ modest adherence	18/85	0·68	0·283, 1·614	0·378	0·73	0·297, 1·776	0·483	0·74	0·297, 1·826	0·509
	Q_4_ high adherence	16/85	0·86	0·342, 2·180	0·756	0·90	0·348, 2·347	0·834	0·94	0·361, 2·464	0·905
	Q_5_ very high adherence	22/85	Ref.								
	High severity (T_3_) *v*. low severity (T_1_)										
	Q_1_ very low adherence	22/87	3·57	1·252, 10·193	**0·02**	4·98	1·571, 15·803	**0·006**	3·46	1·029, 11·656	**0·05**
	Q_2_ low adherence	18/87	3·17	1·077, 9·308	**0·04**	3·55	1·153, 10·952	**0·03**	2·49	0·755, 8·210	0·134
	Q_3_ modest adherence	19/87	1·74	0·642, 4·736	0·275	1·85	0·659, 5·181	0·244	1·83	0·622, 5·400	0·272
	Q_4_ high adherence	19/87	2·51	0·891, 7·058	0·082	2·96	1·013, 8·665	**0·05**	3·18	1·043, 9·682	**0·04**
	Q_5_ very high adherence	9/87	Ref.								
hPDI quintiles	Increased severity (T_2_) *v*. low severity (T_1_)										
	Q_1_ very low adherence	21/85	2·10	0·799, 5·517	0·132	1·95	0·652, 5·812	0·233	1·29	0·415, 4·023	0·659
	Q_2_ low adherence	14/85	1·00	0·384, 2·602	1·000	1·02	0·367, 2·831	0·971	0·57	0·185, 1·739	0·321
	Q_3_ modest adherence	18/85	1·20	0·481, 2·993	0·696	1·12	0·428, 2·934	0·817	0·79	0·289, 2·153	0·644
	Q_4_ high adherence	11/85	0·50	0·195, 1·284	0·150	0·47	0·178, 1·232	0·124	0·37	0·137, 0·998	**0·05**
	Q_5_ very high adherence	21/85	Ref.								
	High severity (T_3_) *v*. low severity (T_1_)										
	Q_1_ very low adherence	20/88	3·50	1·238, 9·891	**0·02**	4·04	1·251, 13·064	**0·02**	2·08	0·592, 7·281	0·254
	Q_2_ low adherence	20/88	2·50	0·934, 6·692	0·068	3·01	1·038, 8·711	**0·04**	1·24	0·373, 4·110	0·728
	Q_3_ modest adherence	20/88	2·33	0·880, 6·188	0·089	2·54	0·905, 7·134	0·077	1·58	0·527, 4·751	0·413
	Q_4_ high adherence	16/88	1·27	0·488, 3·317	0·622	1·15	0·428, 3·112	0·778	0·78	0·271, 2·258	0·651
	Q_5_ very high adherence	12/88	Ref.								
uPDI quintiles	Increased severity (T_2_) *v*. low severity (T_1_)										
	Q_1_ very low adherence	19/85	0·81	0·301, 2·201	0·686	0·82	0·281, 2·407	0·722	1·02	0·335, 3·109	0·971
	Q_2_ low adherence	13/85	0·33	0·122, 0·869	**0·03**	0·33	0·118, 0·929	**0·04**	0·38	0·133, 1·108	0·077
	Q_3_ modest adherence	16/85	0·64	0·234, 1·747	0·384	0·68	0·235, 1·943	0·468	0·61	0·208, 1·808	0·375
	Q_4_ high adherence	17/85	0·57	0·214, 1·503	0·254	0·55	0·200, 1·499	0·241	0·55	0·196, 1·540	0·254
	Q_5_ very high adherence	20/85	Ref.								
	High severity (T_3_) *v*. low severity (T_1_)										
	Q_1_ very low adherence	15/87	0·58	0·212, 1·609	0·298	0·55	0·182, 1·664	0·290	0·98	0·299, 3·195	0·970
	Q_2_ low adherence	11/87	0·24	0·092, 0·681	**0·007**	0·25	0·085, 0·716	**0·01**	0·34	0·107, 1·068	0·065
	Q_3_ modest adherence	23/87	0·84	0·321, 2·180	0·715	0·85	0·303, 2·359	0·749	0·86	0·286, 2·565	0·782
	Q_4_ high adherence	16/87	0·49	0·183, 1·284	0·145	0·45	0·164, 1·253	0·127	0·53	0·179, 1·571	0·252
	Q_5_ very high adherence	22/87	Ref.								

The results of the multinomial regression were expressed as OR with 95 % CI.

MDS, Mediterranean diet score; DASH, Dietary Approaches to Stop Hypertension; oPDI, original Plant-based Diet Index; hPDI, healthy Plant-based Diet Index; uPDI, unhealthy Plant-based Diet Index.

The reference categories for the diet quality indices were ‘very high adherence’ (DASH and PDI) and ‘high adherence’ (MDS).

Confounder adjustments: model VI = age (continuous), sex (male/female) and smoking (yes/no), Alcohol Use Disorders Identification Test Consumption score (continuous), energy kcal/d (continuous) and psychological morbidity (yes/no).

Model V = model VI and BMI (continuous).

sa-SPI tertiles: T_1_ (low severity) ≤ 7; T_2_ (increasing severity) 8–17; T_3_ (high severity) ≥ 18.

MDS tertiles: T_1_ (low adherence) ≤ 3; T_2_ (modest adherence) 4–5; T_3_ (high adherence) ≥ 6.

DASH quintiles = Q_1_ (very low adherence) ≤ 16; Q_2_ (low adherence) 17–20; Q_3_ (modest adherence) 21–24; Q_4_ (high adherence) 25–27; Q_5_ (very high adherence) ≥ 28.

oPDI quintiles = Q_1_ (very low adherence) ≤ 43; Q_2_ (low adherence) 44–47; Q_3_ (modest adherence) 48–51; Q_4_ (high adherence) 52–55; Q_5_ (very high adherence) ≥ 56.

hPDI quintiles = Q_1_ (very low adherence) ≤ 41; Q_2_ (low adherence) 42–47; Q_3_ (modest adherence) 48–52; Q_4_ (high adherence) 53–57; Q_5_ (very high adherence) ≥ 58.

uPDI quintiles = Q_1_ (very low adherence) ≤ 41; Q_2_ (low adherence) 42–48; Q_3_ (modest adherence) 49–51; Q_4_ (high adherence) 52–56; Q_5_ (very high adherence) ≥ 57.

The bold face is for visual indication between significant and non-significant values.

The mediation analysis (online Supplementary Information 9) showed that BMI fully mediated the association between the hPDI, oPDI, uPDI and MDS and psoriasis severity but partially mediated the inverse association with the DASH, indicating an independent association between the DASH diet and psoriasis severity that is not dependent on BMI.

### Dietary score components driving associations between the Mediterranean diet score and the Dietary Approaches to Stop Hypertension score and psoriasis severity

The results of the secondary analysis on the MDS and DASH components are presented in Table 4 and online Supplementary Information 10.

**Table 4.** Extracted DASH and MDS components as standardised predictors of psoriasis severity, followed by the results of the univariate regression analyses adjusted for covariate models I–V

Component	*β*	*P*	*R* ^*2*^	*t*
Red and processed meat (DASH)	0·209	**0·001**	0·059	3·328
Unadjusted	0·254	**< 0·001**		
Model I	0·274	**< 0·001**		
Model II	0·273	**< 0·001**		
Model III	0·289	**< 0·001**		
Model IV	0·288	**< 0·001**		
Model V	0·190	**0·004**		
Nuts and legumes (DASH)	–0·153	**0·02**	0·081	–2·423
Unadjusted	–0·207	**< 0·001**		
Model I	–0·213	**< 0·001**		
Model II	–0·214	**< 0·001**		
Model III	–0·244	**< 0·001**		
Model IV	–0·223	**0·001**		
Model V	–0·128	0·06		
Meat and poultry (MDS)	0·154	**0·02**	0·056	2·482
Unadjusted	0·167	**0·008**		
Model I	0·191	**0·003**		
Model II	0·192	**0·003**		
Model III	0·212	**0·002**		
Model IV	0·208	**0·002**		
Model V	0·147	**0·03**		
Fruits and nuts (MDS)	–0·136	**0·04**	0·072	–2·077
Unadjusted	–0·182	**0·004**		
Model I	–0·178	**0·006**		
Model II	–0·183	**0·005**		
Model III	–0·206	**0·003**		
Model IV	–0·182	**0·008**		
Model V	–0·079	0·24		
Legumes (MDS)	–0·134	**0·04**	0·035	–2·054
Unadjusted	–0·182	**0·004**		
Model I	–0·186	**0·004**		
Model II	–0·186	**0·004**		
Model III	–0·204	**0·002**		
Model IV	–0·192	**0·004**		
Model V	–0·119	0·06		

MDS, Mediterranean diet score; DASH, Dietary Approaches to Stop Hypertension.

Stepwise multiple linear regression values are expressed as standardised *β*-coefficients, *P* values, *R* ^*2*^ values and *t* values.

Univariate linear regression models adjusted for age (continuous), sex (male/female) and smoking (yes/no) (model I), model I and Alcohol Use Disorders Identification Test Consumption score (continuous) (model II), model II and energy kcal/d (continuous) (model III), model III and psychological morbidity (yes/no) (model IV) and model IV and BMI (continuous) (model V).

The bold face is for visual indication between significant and non-significant values.

The red and processed meat component of the DASH score was associated with psoriasis severity (*R* ^*2*^ = 0·059, *β* = 0·209, *t*= 3·328, *P* = 0·001), with greater intakes predicting more severe psoriasis. Likewise, the meat and poultry component of the MDS was positively associated with psoriasis severity (*R* ^*2*^ = 0·056, *β* = 0·154, *t*= 2·482, *P* = 0·02). Both meat components of the DASH and MDS retained significance across all covariate adjustment models at univariate linear regression (*β* = 0·190, *P* = 0·004) and (*β* = 0·147, *P* = 0·03), respectively, even after adjustment for BMI.

On the other hand, the nuts and legume component of the DASH score was negatively associated with psoriasis severity (*R* ^*2*^ = 0·081, *β* = −0·153, *t*= −2·423, *P*= 0·02), with greater intakes predicting milder psoriasis. Similarly, the fruits and nuts (*R* ^*2*^ = 0·072, *β* = −0·136, *t*= −2·077, *P*= 0·04) and legume components (*R* ^*2*^ = 0·035, *β* = −0·134, *t*= −2·054, *P*= 0·04) of the MDS were significant negative predictors for psoriasis severity. Following univariate linear regression with psoriasis severity, nuts and legumes (DASH) (*β* = −0·128, *P*= 0·06), fruits and nuts (MDS) (*β* = −0·079, *P*= 0·24) and legumes (MDS) (*β* = −0·119, *P*= 0·06) retained significance until adjustment for BMI where the association was no longer significant.

## Discussion

This study aimed to examine associations between diet quality and the likelihood of reporting greater psoriasis severity in UK-based adults. Participants with a lower adherence to healthy dietary patterns such as the DASH, hPDI, oPDI and MDS were at least twice as likely to report the highest psoriasis severity.

These findings contribute important observational data to the very limited evidence base examining the role of nutrition in psoriasis and highlight the need for dietary screening in the care pathway for psoriasis with opportunities for dietary interventions. Prescribing a healthy diet could be considered an accessible and cost-effective strategy to potentially mitigate symptom severity. Research in this field, however, is still in the early stages of development. The APPLE study was the first to examine the DASH and PDI in psoriasis, although these indices have been linked with reduced risks of other inflammatory conditions such as CVD, diabetes and obesity^(21,31–34)^, which are comorbid with psoriasis^(6)^.

Modest adherence to the MD (average score of 5 points out of 9) was associated with an increased likelihood of reporting a higher psoriasis severity relative to the highest adherence (average score of 7 points), which aligns with the results of the NutriNet Santé cohort study^(10)^. Although the NutriNet Santé study involved a larger prospective cohort than the APPLE study, it was conducted in a French population where MD adherence was more likely and lacks generalisability to northern European countries such as the UK where the MD is not the traditional eating pattern. Furthermore, the APPLE study classified psoriasis severity using a validated tool, the sa-SPI, whereas the NutriNet Santé study used a combination of self-rated severity, hospitalisation history and medication use as a proxy for severity levels, which may be less accurate.

We identified fruit, nut and legume intakes as components of the MD that were associated with the likelihood of reporting milder psoriasis. This could be linked to (i) the anti-inflammatory properties of a range of (poly)phenols, micronutrients and fatty acids^(35–37)^ and (ii) the insoluble fibre contents of these foods, which may exert immunomodulatory activity through the synthesis of SCFA upon fermentation by the host microbiota^(38)^. However, in an Italian cohort, olive oil and fish emerged as protective foods for severe psoriasis using the PREDIMED questionnaire^(11)^. These differences may be explained by methodological dissimilarities in dietary assessment and DQI, in addition to geographical factors that may influence climate, availability and accessibility of foods such as olive oil, fruits, vegetables and fish^(39)^. In the UK, for example, fish consumption is below recommendations for most of the population, with average intakes of fish and oily fish in adults (aged 19–64 years) reported at 22 g/d and 8 g/d, respectively^(40)^.

Further investigations exhibited associations between higher intakes of red and processed meats (DASH) and higher psoriasis severity, independently of BMI. Meat-derived metabolites that may influence inflammation may be plausible explanations for this relationship. Advanced glycation end products^(41)^ result from non-enzymatic glycation of macronutrients, notably occurring with high-temperature cooking of red meat. Their accumulation within the serum and skin promotes inflammatory activity^(42)^, with preliminary evidence from a cross-sectional study showing that serum advanced glycation end concentrations were positively associated with psoriasis severity^(43)^.

A further potential mechanism that may relate is trimethylamine-N-oxide, a gut metabolite generated from foods of animal origin^(44)^. A cross-sectional study revealed significant correlations between serum trimethylamine-N-oxide and skin and joint symptom severity in individuals with psoriatic arthritis^(45)^. Meta-analyses have established the links between trimethylamine-N-oxide concentrations and cardiovascular events^(46,47)^, but this research area remains widely unexplored in psoriasis.

Mediation analyses showed that BMI modulated the associations between DQI adherence and risks of severe psoriasis, underscoring the involvement of adiposity as a key mediator of the diet-disease severity relationship^(48,49)^. Excess adipose tissue activates cytokine-synthesising immune cells, which aggravate psoriasis^(50)^. Moderate-quality evidence from a Cochrane review suggests that dietary interventions may reduce BMI and improve psoriasis^(7)^. Specific psoriasis-related weight loss recommendations in the UK are targeted at individuals receiving methotrexate treatment to reduce treatment-associated risk of liver disease^(51)^. Studies have shown that weight loss improves treatment responses^(52,53)^ and weight loss interventions such as reduced caloric intake and increased exercise should be considered in this population group. Not all the total effect of the DASH diet on psoriasis severity was accounted for by BMI in the mediation analysis, suggesting there are independent elements that may be implicated in symptom management such as sodium intake^(54)^.

### Strengths and limitations

The study design comprised validated questionnaire measures to assess diet and psoriasis severity. This study was online and therefore accessible to people throughout the UK. Incomplete survey responses, possibly due to response fatigue, reduced the sample size^(55)^, explaining the wide 95 % CI. A greater proportion of participants with incomplete responses had a higher BMI, which may have resulted in self-selection bias. Self-report questionnaires are exposed to under- or overreporting and prone to recall and social-desirability bias^(56)^. The study was limited to individuals with digital access and who are fluent in the English language. Food component categories, for example, fruit and nuts, assume equal contributing effects from both food groups, masking the independent effects of individual foods. The convenience sampling and homogeneity of the APPLE study population, predominantly comprising white middle-aged females, limit the generalisability of the results across wider psoriasis populations. Interpretation of results such as these should be done with caution and in consideration of other determinants of health such as physical activity level and socio-economic status^(57)^, which were not included in the adjustment models due to exclusion for overreporting and missing values. Causality and temporality between DQI adherence and disease severity cannot be assumed as this is a cross-sectional study within a single point in time. Prospective longitudinal analyses are required to confirm the direction of effects for the reported associations.

### Conclusion

Higher disease severity is more likely to be reported by individuals with low adherence to health-promoting dietary patterns. Modifying diets to align with healthier eating patterns may be beneficial to people with psoriasis and may be helpful for symptom severity.

## Supporting information

Zanesco et al. supplementary material 1 Zanesco et al. supplementary material

Zanesco et al. supplementary material 2 Zanesco et al. supplementary material

Zanesco et al. supplementary material 3 Zanesco et al. supplementary material

Zanesco et al. supplementary material 4 Zanesco et al. supplementary material

Zanesco et al. supplementary material 5 Zanesco et al. supplementary material

Zanesco et al. supplementary material 6 Zanesco et al. supplementary material

Zanesco et al. supplementary material 7 Zanesco et al. supplementary material

Zanesco et al. supplementary material 8 Zanesco et al. supplementary material

Zanesco et al. supplementary material 9 Zanesco et al. supplementary material

## Supplementary material

For supplementary material/s referred to in this article, please visit https://doi.org/10.1017/S0007114525000340.
