# Associations between diet quality indices and psoriasis severity: results from the Asking People with Psoriasis about Lifestyle and Eating (APPLE) cross-sectional study. – CORRIGENDUM

**DOI:** 10.1017/S0007114525103498

**Published:** 2025-06-14

**Authors:** Sylvia Zanesco, Thiviyani Maruthappu, Christopher E.M. Griffiths, Kathryn V. Dalrymple, Rachel Gibson, Wendy L. Hall

This corrigendum affects the Mediterranean Diet Score. Although distributions and correlations were not significantly altered from the data in the original manuscript, the revised manuscript comprises of minor amendments to **Table 2** and **Supplementary Information 6**. In the revised manuscript, the associations between the Mediterranean diet score and psoriasis severity are not statistically significant at multinomial regression level resulting in modifications to ORs, 95% CIs, and *P* values in **Table 3**, and **Supplementary Information 8**. Reference to the Mediterranean Diet Score is removed from **Table 4**, **Supplementary Information 9**, and **Supplementary Information 10**. This correction arises from an incidental finding regarding the ‘meat and poultry’ component of the Mediterranean Diet Score, which was subject to a formula error.

Revised text and Table 2

For the MDS (score range 0-9) the mean (standard deviation) was 4.66 (1.72), for the DASH (score range 8-35) it was 23.88 (5.58), and for the PDI subtypes, the mean scores were 51.30 (7.29) for the oPDI, 52.29 (9.05) for the hPDI, and 51.64 (8.34) for the uPDI (score range 17-85 for all).

Only the uPDI reported no significant correlation with psoriasis severity. The remaining diet quality indices were negatively correlated with psoriasis severity: DASH (*r* −0.258, *P* < 0.001), hPDI (*r* −0.203, *P* = 0.001), MDS *(r* −0.192, *P* = 0.002*)* and oPDI (*r* −0.175, *P* = 0.005).

**Table 2.** The mean (standard deviation) of the DQIs across psoriasis severity categories and Pearson’s correlation coefficients with psoriasis severity

	Psoriasis severity				
	Mild (n 62)	Moderate (n 115)	Severe (n 80)	Overall	*r* *P*
**MDS**	4.87 (1.58)	4.74 (1.81)	4.38 (1.74)	4.66 (1.72)	−0.192 **0.002**
**DASH**	25.04 (5.41)	24.18 (5.47)	22.52 (5.64)	23.88 (5.68)	−0.258 **<0.001**
**oPDI**	52.18 (7.70)	51.78 (7.59)	50.06 (6.42)	51.30 (7.29)	−0.175 **0.005**
**hPDI**	54.40 (8.35)	52.20 (9.34)	50.54 (9.06)	52.29 (9.05)	−0.203 **0.001**
**uPDI**	50.44 (7.95)	51.59 (8.40)	52.92 (8.57)	51.64 (8.34)	0.119 0.059

DQI = Diet Quality Index; MDS = Mediterranean Diet Score; DASH = Dietary Approaches to Stop Hypertension; oPDI = original Plant-based Diet Index; hPDI = healthy Plant-based Diet Index; uPDI = unhealthy Plant-based Diet Index.

The mean (standard deviation) of the DQIs are expressed as normalised values.

Psoriasis severity was determined using the self-assessed Simplified Psoriasis Index.

sa-SPI: ≤ 10 points (mild psoriasis); 10 – 20 points (moderate psoriasis); 20 - 70 points (severe psoriasis).

MDS: ≤ 3 points (low adherence); 3 – 6 points (modest adherence); 6 – 9 points (high adherence).

DASH: ≤ 8 points (very low adherence); 8 – 16 points (low adherence); 16 – 24 points (modest adherence); 24 – 32 points (high adherence); 32 – 40 points (very high adherence).

oPDI: ≤ 17 points (very low adherence); 17 – 34 points (low adherence); 34 – 51 points (modest adherence); 51 – 68 points (high adherence); 68 – 75 points (very high adherence).

hPDI: ≤ 17 points (very low adherence); 17 – 34 points (low adherence); 34 – 51 points (modest adherence); 51 – 68 points (high adherence); 68 – 75 points (very high adherence).

uPDI: ≤ 17 points (very low adherence); 17 – 34 points (low adherence); 34 – 51 points (modest adherence); 51 – 68 points (high adherence); 68 – 75 points (very high adherence).

Revised Supplementary Information 6

**Table:** **Supplementary Information 6.** the mean (standard deviation) for sa-SPI and MDS tertiles, and DASH and PDI quintiles

		Category	*n* (%)	Mean (SD) DQI score	Mean (SD) sa-SPI score
**sa-SPI**	T_1_	Lower severity	83 (32)		6.31 (4.87)
	T_2_	Increasing severity	85 (33)		16.71 (2.36)
	T_3_	Higher severity	88 (35)		27.21 (4.98)
**MDS**	T_1_	Low adherence	75 (29)	2.62 (0.77)	19.67 (10.13)
	T_2_	Modest adherence	97 (38)	4.56 (0.43)	16.20 (8.87)
	T_3_	High adherence	85 (33)	6.59 (0.91)	15.89 (9.74)
**DASH**	Q_1_	Very low adherence	49 (19)	15.95 (2.41)	21.33 (9.61)
	Q_2_	Low adherence	54 (21)	20.79 (0.97)	18.37 (10.00)
	Q_3_	Modest adherence	49 (19)	23.79 (0.74)	16.04 (7.86)
	Q_4_	High adherence	49 (19)	26.61 (0.83)	14.83 (8.66)
	Q_5_	Very high adherence	56 (22)	31.50 (2.55)	14.37 (9.82)
**oPDI**	Q_1_	Very low adherence	53 (21)	41.23 (3.25)	19.71 (11.03)
	Q_2_	Low adherence	42 (16)	47.15 (0.98)	18.67 (8.09)
	Q_3_	Modest adherence	60 (23)	51.05 (1.22)	15.99 (9.92)
	Q_4_	High adherence	51 (20)	55.36 (1.24)	16.07 (8.53)
	Q_5_	Very high adherence	50 (20)	61.62 (3.16)	14.49 (8.91)
**hDPI**	Q_1_	Very low adherence	52 (20)	39.69 (4.01)	18.26 (9.66)
	Q_2_	Low adherence	48 (19)	47.33 (1.43)	19.24 (9.25)
	Q_3_	Modest adherence	53 (21)	52.13 (1.37)	18.20 (9.70)
	Q_4_	High adherence	49 (19)	56.99 (1.48)	15.78 (9.50)
	Q_5_	Very high adherence	54 (21)	64.74 (4.04)	13.63 (8.85)
**uPDI**	Q_1_	Very low adherence	48 (18)	39.62 (3.64)	16.53 (10.00)
	Q_2_	Low adherence	49 (20)	46.72 (1.39)	14.14 (8.76)
	Q_3_	Modest adherence	54 (21)	51.34 (1.28)	17.72 (9.96)
	Q_4_	High adherence	51 (20)	55.89 (1.37)	17.34 (9.93)
	Q_5_	Very high adherence	54 (21)	63.08 (3.71)	18.53 (8.81)

DQI = Diet Quality Index; sa-SPI = self-assessed Simplified Psoriasis Index; MDS = Mediterranean Diet Score; DASH = Dietary Approaches to Stop Hypertension; oPDI = original Plant-based Diet Index; hPDI = healthy Plant-based Diet Index; uPDI = unhealthy Plant-based Diet Index. n 1 excluded from rank transformation for sa-SPI (59 points), n 1 excluded from rank transformation for oPDI (73 points), n 1 excluded from rank transformation for hPDI (76 points), and n 1 excluded from rank transformation for uPDI (73 points).

Revised text and Table 3

When adjusted for age, sex, smoking, AUDIT-C, energy intake, and psychological morbidity, very low adherence to the DASH (OR = 3.75, 95% CI 1.313 – 10.700, *P* = 0.01), and hPDI (OR = 4.04, 95% CI 1.251 – 13.064, *P* = 0.02) was associated with an increased likelihood of reporting higher psoriasis severity relative to very high adherence.

**Table 3.** Diet quality indices and the unadjusted and adjusted OR (95% CI) for psoriasis severity

			unadjusted			Model IV			Model V			
		cases/n	OR	95% CI	*P*	OR	95% CI	*P*	OR	95% CI	*P*	
**MDS** **tertiles**	**Increased severity (T**_**2**_ **) vs low severity (T**_**1**_ **)**											
	T_1_ low adherence	25/91	1.13	0.518 – 2.460	0.760	0.96	0.417 – 2.203	0.919	0.73	0.304 – 1.746	0.478	
	T_2_ modest adherence	35/91	0.96	0.477 – 1.925	0.904	0.84	0.407 – 1.751	0.649	0.73	0.348 – 1.550	0.417	
	T_3_ high adherence	31/91	Ref.									
	**High severity (T**_**3**_ **) vs** **low severity (T**_**1**_ **)**											
	T_1_ low adherence	30/84	1.62	0. 742 – 3.516	0.227	1.94	0.820 – 4.594	0.132	1.00	0.390 – 2.578	0.996	
	T_2_ modest adherence	28/84	0.91	0.439 – 1.903	0.810	1.03	0.464 – 2.264	0.951	0.65	0.277 – 1.517	0.318	
	T_3_ high adherence	26/84	Ref.									
**DASH** **quintiles**	**Increased severity (T**_**2**_ **) vs low severity (T**_**1**_ **)**											
	Q_1_ Very low adherence	15/85	2.03	0.734 – 5.608	0.172	2.03	0.693 – 5.919	0.197	1.27	0.406 – 4.000	0.678	
	Q_2_ Low adherence	13/85	0.98	0.378 – 2.526	0.962	0.93	0.353 – 2.425	0.874	0.66	0.241 – 1.811	0.420	
	Q_3_ Modest adherence	19/85	1.71	0.681 – 4.312	0.253	1.63	0.632 – 4.207	0.312	1.35	0.511 – 3.562	0.545	
	Q_4_ High adherence	21/85	1.67	0.683 – 4.092	0.261	1.68	0.674 – 4.164	0.267	1.51	0.602 – 3.807	0.378	
	Q_5_ Very high adherence	17/85	Ref.									
	**High severity (T**_**3**_ **) vs** **low severity (T**_**1**_ **)**											
	Q_1_ Very low adherence	24/88	3.45	1.301 – 9.150	**0.01**	3.75	1.313 – 10.700	**0.01**	1.70	0.538 – 5.340	0.368	
	Q_2_ Low adherence	23/88	1.84	0.756 – 4.461	0.179	1.81	0.726 – 4.514	0.203	1.04	0.389 – 2.784	0.936	
	Q_3_ Modest adherence	14/88	1.34	0.509 – 3.533	0.552	1.33	0.487 – 3.626	0.578	0.93	0.323 – 2.644	0.884	
	Q_4_ High adherence	11/88	0.93	0.345 – 2.506	0.886	0.98	0.356 – 2.684	0.965	0.77	0.269 – 2.175	0.616	
	Q_5_ Very high adherence	16/88	Ref.									
**oPDI quintiles**	**Increased severity (T**_**2**_ **) vs low severity (T**_**1**_ **)**											
	Q_1_ Very low adherence	17/85	1.13	0.438 – 2.914	0.801	1.40	0.491 – 3.971	0.531	1.14	0.393 – 3.318		0.808
	Q_2_ Low adherence	12/85	0.86	0.315 – 2.368	0.776	0.94	0.331 – 2.679	0.911	0.81	0.280 – 2.352		0.700
	Q_3_ Modest adherence	18/85	0.68	0.283 – 1.614	0.378	0.73	0.297 – 1.776	0.483	0.74	0.297 – 1.826		0.509
	Q_4_ High adherence	16/85	0.86	0.342 – 2.180	0.756	0.90	0.348 – 2.347	0.834	0.94	0.361 – 2.464		0.905
	Q_5_ Very high adherence	22/85	Ref.									
	**High severity (T**_**3**_ **) vs** **low severity (T**_**1**_ **)**											
	Q_1_ Very low adherence	22/87	3.57	1.252 – 10.193	**0.02**	4.98	1.571 – 15.803	**0.006**	3.46	1.029 – 11.656		**0.05**
	Q_2_ Low adherence	18/87	3.17	1.077 – 9.308	**0.04**	3.55	1.153 – 10.952	**0.03**	2.49	0.755 – 8.210		0.134
	Q_3_ Modest adherence	19/87	1.74	0.642 – 4.736	0.275	1.85	0.659 – 5.181	0.244	1.83	0.622 – 5.400		0.272
	Q_4_ High adherence	19/87	2.51	0.891 – 7.058	0.082	2.96	1.013 – 8.665	**0.05**	3.18	1.043 – 9.682		**0.04**
	Q_5_ Very high adherence	9/87	Ref.									
**hPDI** **quintiles**	**Increased severity (T**_**2**_ **) vs low severity (T**_**1**_ **)**											
	Q_1_ Very low adherence	21/85	2.10	0.799 – 5.517	0.132	1.95	0.652 – 5.812	0.233	1.29	0.415 – 4.023	0.659	
	Q_2_ Low adherence	14/85	1.00	0.384 – 2.602	1.000	1.02	0.367 – 2.831	0.971	0.57	0.185 – 1.739	0.321	
	Q_3_ Modest adherence	18/85	1.20	0.481 – 2.993	0.696	1.12	0.428 – 2.934	0.817	0.79	0.289 – 2.153	0.644	
	Q_4_ High adherence	11/85	0.50	0.195 – 1.284	0.150	0.47	0.178 – 1.232	0.124	0.37	0.137 – 0.998	**0.05**	
	Q_5_ Very high adherence	21/85	Ref.									
	**High severity (T**_**3**_ **) vs** **low severity (T**_**1**_ **)**											
	Q_1_ Very low adherence	20/88	3.50	1.238 – 9.891	**0.02**	4.04	1.251 – 13.064	**0.02**	2.08	0.592 – 7.281	0.254	
	Q_2_ Low adherence	20/88	2.50	0.934 – 6.692	0.068	3.01	1.038 – 8.711	**0.04**	1.24	0.373 – 4.110	0.728	
	Q_3_ Modest adherence	20/88	2.33	0.880 – 6.188	0.089	2.54	0.905 – 7.134	0.077	1.58	0.527 – 4.751	0.413	
	Q_4_ High adherence	16/88	1.27	0.488 – 3.317	0.622	1.15	0.428 – 3.112	0.778	0.78	0.271 – 2.258	0.651	
	Q_5_ Very high adherence	12/88	Ref.									
**uPDI** **quintiles**	**Increased severity (T**_**2**_ **) vs low severity (T**_**1**_ **)**											
	Q_1_ Very low adherence	19/85	0.81	0.301 – 2.201	0.686	0.82	0.281 – 2.407	0.722	1.02	0.335 – 3.109	0.971	
	Q_2_ Low adherence	13/85	0.33	0.122 – 0.869	**0.03**	0.33	0.118 – 0.929	**0.04**	0.38	0.133 – 1.108	0.077	
	Q_3_ Modest adherence	16/85	0.64	0.234 – 1.747	0.384	0.68	0.235 – 1.943	0.468	0.61	0.208 – 1.808	0.375	
	Q_4_ High adherence	17/85	0.57	0.214 – 1.503	0.254	0.55	0.200 – 1.499	0.241	0.55	0.196 – 1.540	0.254	
	Q_5_ Very high adherence	20/85	Ref.									
	**High severity (T**_**3**_ **) vs** **low severity (T**_**1**_ **)**											
	Q_1_ Very low adherence	15/87	0.58	0.212 – 1.609	0.298	0.55	0.182 – 1.664	0.290	0.98	0.299 – 3.195	0.970	
	Q_2_ Low adherence	11/87	0.24	0.092 – 0.681	**0.007**	0.25	0.085 – 0.716	**0.01**	0.34	0.107 – 1.068	0.065	
	Q_3_ Modest adherence	23/87	0.84	0.321 – 2.180	0.715	0.85	0.303 – 2.359	0.749	0.86	0.286 – 2.565	0.782	
	Q_4_ High adherence	16/87	0.49	0.183 – 1.284	0.145	0.45	0.164 – 1.253	0.127	0.53	0.179 – 1.571	0.252	
	Q_5_ Very high adherence	22/87	Ref.									

Results of the multinomial regression were expressed as Odds Ratios (OR) with 95% Confidence Intervals (CI).

MDS = Mediterranean Diet Score; DASH = Dietary Approaches to Stop Hypertension; oPDI = original Plant-based Diet Index; hPDI = healthy Plant-based Diet Index; uPDI = unhealthy Plant-based Diet Index.

The reference categories for the diet quality indices were “very high adherence” (DASH and PDIs) and “high adherence” (MDS).

Confounder adjustments: Model VI = age (continuous), sex (male/female) and smoking (yes/no), Alcohol Use Disorders Identification Test Consumption score (continuous), energy kcal/day (continuous), and psychological morbidity (yes/no).

Model V = model VI and body mass index (continuous).

sa-SPI tertiles: T_1_ (low severity) ≤ 7; T_2_ (increasing severity) 8 - 17; T_3_ (high severity) ≥ 18.

MDS tertiles: T_1_ (low adherence) ≤ 3; T_2_ (modest adherence) 4 - 5; T_3_ (high adherence) ≥ 6.

DASH quintiles = Q_1_ (very low adherence) ≤ 16; Q_2_ (low adherence) 17 - 20; Q_3_ (modest adherence) 21 - 24; Q_4_ (high adherence) 25 - 27; Q_5_ (very high adherence) ≥ 28.

oPDI quintiles = Q_1_ (very low adherence) ≤ 43; Q_2_ (low adherence) 44 - 47; Q_3_ (modest adherence) 48 - 51; Q_4_ (high adherence) 52 - 55; Q_5_ (very high adherence) ≥ 56.

hPDI quintiles = Q_1_ (very low adherence) ≤ 41; Q_2_ (low adherence) 42 - 47; Q_3_ (modest adherence) 48 - 52; Q_4_ (high adherence) 53 - 57; Q_5_ (very high adherence) ≥ 58.

uPDI quintiles = Q_1_ (very low adherence) ≤ 41; Q_2_ (low adherence) 42 - 48; Q_3_ (modest adherence) 49 - 51; Q_4_ (high adherence) 52 - 56; Q_5_ (very high adherence) ≥ 57.

Revised Supplementary Information 8

**Table:** **Supplementary Information 8.** diet quality indices and the adjusted OR (95% CI) for psoriasis severity (Model I-III)

			Model I			Model II			Model lII		
		cases/n	OR	95% CI	*P*	OR	95% CI	*P*	OR	95% CI	*P*
**MDS** **tertiles**	**Increased severity (T**_**2**_ **) vs low severity (T**_**1**_ **)**										
	T_1_ low adherence	25/91	0.96	0.423 – 2.162	0.915	0.95	0.417 – 2.157	0.901	0.96	0.419 – 2.212	0.929
	T_2_ modest adherence	35/91	0.84	0.408 – 1.723	0.631	0.83	0.403 – 1.715	0. 617	0.84	0.405 – 1.745	0.641
	T_3_ high adherence	31/91	Ref.								
	**High severity (T**_**3**_ **) vs low severity (T**_**1**_ **)**										
	T_1_ low adherence	30/84	1.82	0.794 – 4.181	0.157	1.88	0.808 – 4.347	0.143	1.99	0.848 – 4681	0.113
	T_2_ modest adherence	28/84	0.94	0.435 – 2.049	0.884	0.96	0.441 – 2.094	0.921	1.00	0.458 – 2.201	0.993
	T_3_ high adherence	26/84	Ref.								
**DASH** **quintiles**	**Increased severity (T**_**2**_ **) vs low severity (T**_**1**_ **)**										
	Q_1_ Very low adherence	15/85	2.04	0.703 – 5.921	0.189	2.06	0.707 – 5.970	0.186	2.07	0.710 – 6.007	0.183
	Q_2_ Low adherence	13/85	0.91	0.350 – 2.388	0.855	0.92	0.350 – 2.391	0.856	0.94	0.357 – 2.453	0.894
	Q_3_ Modest adherence	19/85	1.59	0.623 – 4.055	0.332	1.62	0.630 – 4.160	0.317	1.65	0.641 – 4.255	0.299
	Q_4_ High adherence	21/85	1.64	0.661 – 4.045	0.288	1.65	0.665 – 4.084	0.281	1.68	0.674 – 4.166	0.266
	Q_5_ Very high adherence	17/85	Ref.								
	**High severity (T**_**3**_ **) vs low severity (T**_**1**_ **)**										
	Q_1_ Very low adherence	24/88	4.09	1.455 – 11.494	**0.008**	4.10	1.457 – 11.539	**0.008**	4.10	1.458 – 11.547	**0.008**
	Q_2_ Low adherence	23/88	1.87	0.757 – 4.604	0.175	1.87	0.758 – 4.606	0.175	1.89	0.764 – 4.661	0.169
	Q_3_ Modest adherence	14/88	1.38	0.514 – 3.693	0.524	1.39	0.513 – 3.736	0.520	1.40	0.518 – 3.779	0.508
	Q_4_ High adherence	11/88	0.96	0.351 – 2.595	0.928	0.96	0.352 – 2.603	0.931	0.97	0.354 – 2.629	0.944
	Q_5_ Very high adherence	16/88	Ref.								
**oPDI** **quintiles**	**Increased severity (T**_**2**_ **) vs low severity (T**_**1**_ **)**										
	Q_1_ Very low adherence	17/85	1.19	0.445 – 3.162	0.733	1.19	0.445 – 3.162	0.733	1.42	0.498 – 4.034	0.514
	Q_2_ Low adherence	12/85	0.84	0.305 – 2.317	0.736	0.84	0.304 – 2.332	0.741	0.94	0.331 – 2.665	0.906
	Q_3_ Modest adherence	18/85	0.68	0.282 – 1.619	0.379	0.68	0.282 – 1.623	0.381	0.74	0.301 – 1.797	0.500
	Q_4_ High adherence	16/85	0.86	0.336 – 2.212	0.758	0.87	0.335 – 2.230	0.763	0.89	0.344 – 2.317	0.816
	Q_5_ Very high adherence	22/85	Ref.								
	**High severity (T**_**3**_ **) vs low severity (T**_**1**_ **)**										
	Q_1_ Very low adherence	22/87	4.09	1.391 – 12.019	**0.01**	4.09	1.391 – 12.020	**0.01**	5.10	1.628 – 15.954	**0.005**
	Q_2_ Low adherence	18/87	3.06	1.035 – 9.025	**0.04**	3.07	1.034 – 9.107	**0.04**	3.52	1.155 – 10.700	**0.03**
	Q_3_ Modest adherence	19/87	1.72	0.629 – 4.675	0.292	1.72	0.630 – 4.689	0.291	1.90	0.686 – 5.285	0.216
	Q_4_ High adherence	19/87	2.70	0.944 – 7.715	0.064	2.71	0.943 – 7.788	0.064	2.81	0.972 – 8.115	0.057
	Q_5_ Very high adherence	9/87	Ref.								
**hPDI** **quintiles**	**Increased severity (T**_**2**_ **) vs low severity (T**_**1**_ **)**										
	Q_1_ Very low adherence	21/85	2.09	0.746 – 5.828	0.161	2.06	0.732 – 5.770	0.171	2.00	0.678 – 5.924	0.209
	Q_2_ Low adherence	14/85	1.06	0.386 – 2.882	0.918	1.05	0.385 – 2.877	0.921	1.04	0.375 – 2.875	0.942
	Q_3_ Modest adherence	18/85	1.16	0.451 – 2.979	0.760	1.15	0.448 – 2.968	0.767	1.14	0.436 – 2.973	0.792
	Q_4_ High adherence	11/85	0.49	0.191 – 1.276	0.145	0.48	0.186 – 1.262	0.138	0.48	0.185 – 1.258	0.136
	Q_5_ Very high adherence	21/85	Ref.								
	**High severity (T**_**3**_ **) vs low severity (T**_**1**_ **)**										
	Q_1_ Very low adherence	20/88	4.31	1.429 – 12.984	**0.009**	4.28	1.416 – 12.951	**0.01**	4.69	1.468 – 14.967	**0.009**
	Q_2_ Low adherence	20/88	3.14	1.110 – 8.850	**0.03**	3.13	1.109 – 8.842	**0.03**	3.28	1.145 – 9.415	**0.03**
	Q_3_ Modest adherence	20/88	2.63	0.958 – 7.193	0.061	2.62	0.956 – 7.181	0.061	2.75	0.986 – 7.675	**0.05**
	Q_4_ High adherence	16/88	1.30	0.490 – 3.396	0.606	1.28	0.482 – 3.393	0.621	1.30	0.490 – 3.471	0.595
	Q_5_ Very high adherence	12/88	Ref.								
**uPDI** **quintiles**	**Increased severity (T**_**2**_ **) vs low severity (T**_**1**_ **)**										
	Q_1_ Very low adherence	19/85	0.86	0.299 – 2.468	0.777	0.86	0.299 – 2.469	0.777	0.81	0.277 – 2.351	0.695
	Q_2_ Low adherence	13/85	0.33	0.120 – 0.929	**0.04**	0.33	0.120 – 0.930	**0.04**	0.32	0.116 – 0.907	**0.03**
	Q_3_ Modest adherence	16/85	0.66	0.232 – 1.883	0.438	0.66	0.232 – 1.888	0.440	0.66	0.231 – 1.885	0.437
	Q_4_ High adherence	17/85	0.53	0.196 – 1.455	0.220	0.54	0.196 – 1.459	0.222	0.54	0.197 – 1.465	0.225
	Q_5_ Very high adherence	20/85	Ref.								
	**High severity (T**_**3**_ **) vs low severity (T**_**1**_ **)**										
	Q_1_ Very low adherence	15/87	0.51	0.172 – 1.497	0.219	0.51	0.172 – 1.497	0.219	0.49	0.164 – 1.457	0.199
	Q_2_ Low adherence	11/87	0.23	0.079 – 0.642	**0.005**	0.23	0.079 – 0.642	**0.005**	0.22	0.077 – 0.632	**0.005**
	Q_3_ Modest adherence	23/87	0.76	0.275 – 2.069	0.585	0.76	0.275 – 2.070	0.585	0.75	0.274 – 2.069	0.583
	Q_4_ High adherence	16/87	0.42	0.152 – 1.133	0.086	0.42	0.152 – 1.133	0.086	0.42	0.152 – 1.136	0.087
	Q_5_ Very high adherence	22/87	Ref.								

Results of the multinomial regression were expressed as Odds Ratios (OR) with 95% Confidence Intervals (CI).

MDS = Mediterranean Diet Score; DASH = Dietary Approaches to Stop Hypertension; oPDI = original Plant-based Diet Index; hPDI = healthy Plant-based Diet Index; uPDI = unhealthy Plant-based Diet Index.

The reference categories for the diet quality indices were “very high adherence” (DASH and PDIs) and “high adherence” (MDS).

Confounder adjustments.

Model I = age (continuous), sex (male/female) and smoking (yes/no).

Model II = Model I and Alcohol Use Disorders Identification Test Consumption score (continuous).

Model III = Model II and energy kcal/day (continuous).

sa-SPI tertiles: T_1_ (low severity) ≤ 7; T_2_ (increasing severity) 8 - 17; T_3_ (high severity) ≥ 18.

MDS tertiles: T_1_ (low adherence) ≤ 3; T_2_ (modest adherence) 4 - 5; T_3_ (high adherence) ≥ 6.

DASH quintiles = Q_1_ (very low adherence) ≤ 16; Q_2_ (low adherence) 17 - 20; Q_3_ (modest adherence) 21 - 24; Q_4_ (high adherence) 25 - 27; Q_5_ (very high adherence) ≥ 28.

oPDI quintiles = Q_1_ (very low adherence) ≤ 43; Q_2_ (low adherence) 44 - 47; Q_3_ (modest adherence) 48 - 51; Q_4_ (high adherence) 52 - 55; Q_5_ (very high adherence) ≥ 56.

hPDI quintiles = Q_1_ (very low adherence) ≤ 41; Q_2_ (low adherence) 42 - 47; Q_3_ (modest adherence) 48 - 52; Q_4_ (high adherence) 53 - 57; Q_5_ (very high adherence) ≥ 58.

uPDI quintiles = Q_1_ (very low adherence) ≤ 41; Q_2_ (low adherence) 42 - 48; Q_3_ (modest adherence) 49 - 51; Q_4_ (high adherence) 52 - 56; Q_5_ (very high adherence) ≥ 57.

Revised text and Table 4

The red and processed meat component of the DASH score was associated with psoriasis severity (*R* ^*2*^ *0.059, β = 0.209, t = 3.328, P = 0.001),* with greater intakes predicting more severe psoriasis and retained significance across all covariate adjustment models in the univariate linear regression (*β = 0.190, P = 0.004)*, even after adjustment for BMI. On the other hand, the nuts and legume component of the DASH score was negatively associated with psoriasis severity (*R* ^*2*^ *0.081, β = -0.153, t = −2.423, P = 0.02)* with greater intakes predicting milder psoriasis and retained significance until adjustment for BMI where the association was no longer significant *(β = −0.128, P = 0.06).*

**Table 4.** Extracted DASH components as standardised predictors of psoriasis severity, followed by the results of the univariate regression analyses adjusted for covariate models I-V

Component	*β*	*P values*	*R* ^*2*^	*t*
Red and processed meat	0.209	**0.001**	0.059	3.328
unadjusted	0.254	**<0.001**		
Model I	0.274	**<0.001**		
Model II	0.273	**<0.001**		
Model III	0.289	**<0.001**		
Model IV	0.288	**<0.001**		
Model V	0.190	**0.004**		
Nuts and legumes	−0.153	**0.02**	0.081	−2.423
unadjusted	−0.207	**<0.001**		
Model I	−0.213	**<0.001**		
Model II	−0.214	**<0.001**		
Model III	−0.244	**<0.001**		
Model IV	−0.223	**0.001**		
Model V	−0.128	0.06		

DASH = Dietary Approaches to Stop Hypertension.

Stepwise multiple linear regression values are expressed as standardised *β*-coefficients, *P* values, R^2^ values and *t* values.

Univariate linear regression models adjusted for age (continuous), sex (male/female) and smoking (yes/no)(model I), model I and Alcohol Use Disorders Identification Test Consumption score (continuous)(model II), model II and energy kcal/day (continuous) (model III), model III and psychological morbidity (yes/no) (model IV), and model IV and body mass index (continuous) (model V).

Revised text and Supplementary Information 9

The mediation analysis (**Supplementary Information 9**) showed that BMI fully mediated the association between the hPDI, oPDI, and uPDI, and psoriasis severity, but partially mediated the inverse association with the DASH indicating an independent association between the DASH diet and psoriasis severity that is not dependent on BMI.

**Table:** **Supplementary Information 9.** mediation analysis examining the mediating effect of BMI on the DQI and psoriasis severity associations

	Unstandardised - *β*	95% CI	*P*	Model tested
DASH → BMI	−0.476	−0.604 – −0.348	**<0.001**	Exposure-Mediator
DASH + BMI → sa-SPI				
DASH	−0.226	−0.443 – −0.009	**0.04**	Direct effect
BMI	0.457	0.265 – 0.650	**<0.001**	
DASH → sa-SPI	−0.440	−0.644 – −0.236	**<0.001**	Total effect
oPDI → BMI	−0.189	−0.294 – −0.083	**<0.001**	Exposure-Mediator
oPDI + BMI → sa-SPI				
oPDI	−0.132	−0.288 – 0.023	0.094	Direct effect
BMI	0.512	0.333 – 0.691	**<0.001**	
oPDI → sa-SPI	−0.229	−0.389 – −0.069	**0.005**	Total effect
hPDI → BMI	−0.267	−0.348 – 0.187	**<0.001**	Exposure-Mediator
hPDI + BMI → sa-SPI				
hPDI	−0.089	−0.221 – 0.044	0.188	Direct effect
BMI	0.494	0.304 – 0.683	**<0.001**	
hPDI → sa-SPI	−0.215	−0.343 – 0.087	**0.001**	Total effect
uPDI → BMI	0.194	0.103 – 0.286	**<0.001**	Exposure-Mediator
uPDI + BMI → sa-SPI				
uPDI	0.029	−0.109 – 0.168	0.679	Direct effect
BMI	0.531	0.348 – 0.713	**<0.001**	
uPDI → sa-SPI	0.136	−0.005 – 0.276	0.06	Total effect

BMI = Body Mass Index; DQI = Diet Quality Index; sa-SPI = self-assessed Simplified Psoriasis Index; DASH = Dietary Approaches to Stop Hypertension; oPDI = original Plant-based Diet Index; hPDI = healthy Plant-based Diet Index; uPDI = unhealthy Plant-based Diet Index.

A 3-step mediation analysis was conducted. Step 1 = linear regression with DQI (as the independent variable) and BMI (as the dependent variable) for the exposure-mediator effect. Step 2 = a multiple linear regression with DQI and BMI (as the independent variables) and sa-SPI (as the dependent variable) for the direct effect. Step 3 = linear regression with DQI (as the independent variable) and sa-SPI (as the dependent variable) for the total effect. Partial mediation = if all unstandardised - *β* coefficients for a given DQI are statistically significant and the unstandardised - *β* coefficient of the DQI in the direct effect model is closer to zero than that of the DQI in the total effect model. Full mediation = if the unstandardised - *β* coefficient for a given DQI is not statistically significant within the direct effect model, but the mediator is statistically significant. The effect of the oPDI, and hPDI on sa-SPI is fully mediated by BMI and disappears when the BMI is added to the regression model. BMI partially mediates the effect of the DASH on sa-SPI meaning that when BMI is added to the model, the effect of DASH is reduced but not nullified.

Revised Supplementary Information 10

**Table:** **Supplementary Information 10.** extracted DASH components as unstandardised predictors of psoriasis severity, followed by the results of the univariate regression analyses adjusted for covariate models I-V

Component	*β*	*P values*	*R* ^*2*^		*t*
Red and processed meat	0.050	**0.001**	0.059	3.328	
unadjusted	0.061 (0.032-0.089)	**<0.001**			
Model I	0.065 (0.036-0.094)	**<0.001**			
Model II	0.065 (0.036-0.094)	**<0.001**			
Model III	0.069 (0.039-0.099)	**<0.001**			
Model IV	0.068 (0.039-0.098)	**<0.001**			
Model V	0.045 (0.015-0.076)	**0.004**			
Nuts and legumes	−0.019	**0.02**	0.081	-2.423	
unadjusted	−0.026 (-0.042,-0.011)	**<0.001**			
Model I	−0.027 (-0.043,-0.011)	**<0.001**			
Model II	−0.027 (-0.043,-0.011)	**<0.001**			
Model III	−0.031 (-0.048,-0.014)	**<0.001**			
Model IV	−0.028 (-0.085,0.097)	**0.001**			
Model V	−0.016 (-0.033,0.001)	0.06			


DASH = Dietary Approaches to Stop Hypertension.

Stepwise multiple linear regression values are expressed as unstandardised *β*-coefficients (95% confidence intervals), *P* values, R^2^ values and t values.

Univariate linear regression models adjusted for age (continuous), sex (male/female) and smoking (yes/no)(model I), model I and Alcohol Use Disorders Identification Test Consumption score (continuous)(model II), model II and energy kcal/day (continuous) (model III), model III and psychological morbidity (yes/no) (model IV), and model IV and body mass index (continuous) (model V).

Revised text in discussion

Participants with a lower adherence to healthy dietary patterns such as the DASH, hPDI, and oPDI, were at least twice as likely to report the highest psoriasis severity.

Adherence to the MD was inversely correlated with psoriasis severity but was not a predictor of disease severity, conflicting with the results of the NutriNet Santé cohort study [10]. The NutriNet Santé study involved a larger prospective cohort than the APPLE study, and was conducted in a French population where MD adherence was more likely and lacks generalisability to northern European countries such as the UK where the MD is not the traditional eating pattern. The APPLE study however, classified psoriasis severity using a validated tool, the sa-SPI, whereas the NutriNet Santé study used a combination of self-rated severity, hospitalisation history, and medication use as a proxy for severity levels, which may be less accurate.

We identified the nuts and legume component of the DASH score that was associated with likelihood of reporting milder psoriasis. This could be linked to: (i) the anti-inflammatory properties of a range of (poly)phenols, micronutrients, and fatty acids [35-37], and (ii) the insoluble fibre contents of these foods, which may exert immunomodulatory activity through the synthesis of short chain fatty acids upon fermentation by the host microbiota [38]. In an Italian cohort, olive oil and fish emerged as protective foods for severe psoriasis as per the PREDIMED questionnaire [11].

