# Supplementary material for: Associations between diet quality indices and psoriasis severity: results from the Asking People with Psoriasis about Lifestyle and Eating (APPLE) cross-sectional study
Source: Br J Nutr. 2025 Feb 20;133(4):546–57. doi: 10.1017/S0007114525000340 (PMC12011542; doi:10.1017/S0007114525000340)
Supplement: Zanesco et al. supplementary material 1 — Zanesco et al. supplementary material [file S0007114525000340sup001.docx]

| **Supplementary Information 10.** Extracted DASH and MDS components as unstandardised predictors of psoriasis severity, followed by the results of the univariate regression analyses adjusted for covariate models I-V. | | | | | | | |  |
| --- | --- | --- | --- | --- | --- | --- | --- | --- |
| Component | *β* | *P values* | *R^2^* | | | | *t* |  |
| Red and processed meat (DASH) | 0.050 | **0.001** | 0.059 | | | 3.328 | |  |
| unadjusted | 0.061  (0.032-0.089) | **<0.001** |  | | |  | |  |
| Model I | 0.065  (0.036-0.094) | **<0.001** |  | | |  | |  |
| Model II | 0.065  (0.036-0.094) | **<0.001** |  | | |  | |  |
| Model III | 0.069  (0.039-0.099) | **<0.001** |  | | |  | |  |
| Model IV | 0.068  (0.039-0.098) | **<0.001** |  | | |  | |  |
| Model V | 0.045  (0.015-0.076) | **0.004** |  | | |  | |  |
| Nuts and Legumes (DASH) | -0.019 | **0.02** | 0.081 | | | -2.423 | |  |
| unadjusted | -0.026  (-0.042,-0.011) | **<0.001** |  | | | | |  |
| Model I | -0.027  (-0.043,-0.011) | **<0.001** |  | | | | |  |
| Model II | -0.027  (-0.043,-0.011) | **<0.001** |  | | | | |  |
| Model III | -0.031  (-0.048,-0.014) | **<0.001** |  | | | | |  |
| Model IV | -0.028  (-0.085,0.097) | **0.001** |  | | | | |  |
| Model V | -0.016  (-0.033,0.001) | 0.06 |  | | | | |  |
| Meat and poultry (MDS) | 0.047 | **0.02** | 0.056 | 2.482 | | | |  |
| unadjusted | 0.050  (0.013,0.088) | **0.008** |  |  | | | |  |
| Model I | 0.058  (0.020,0.095) | **0.003** |  |  | | | |  |
| Model II | 0.058  (0.020,0.096) | **0.003** |  | | | | |  |
| Model III | 0.064  (0.024,0.105) | **0.002** |  | | | | |  |
| Model IV | 0.063  (0.023,0.103) | **0.002** |  | | | | |  |
| Model V | 0.044  (0.006,0.083) | **0.03** |  | | | | |  |
| Fruits and nuts (MDS) | -0.006 | **0.04** | 0.072 | -2.077 | | | |  |
| unadjusted | -0.008  (-0.013,-0.003) | **0.004** |  | | | | |  |
| Model I | -0.008  (-0.013,-0.002) | **0.006** |  | | | | |  |
| Model II | -0.008  (-0.014,-0.002) | **0.005** |  | | | | |  |
| Model III | -0.009  (-0.015,-0.003) | **0.003** |  | | | | |  |
| Model IV | -0.008  (-0.014,-0.002) | **0.008** |  | | | | |  |
| Model V | -0.004  (-0.009,0.002) | 0.24 |  | | | | |  |
| Legumes (MDS) | -0.019 | **0.04** | 0.035 | | -2.054 | | |  |
| unadjusted | -0.026  (-0.043,-0.008) | **0.004** |  | | | | |  |
| Model I | -0.027  (-0.044,-0.009) | **0.004** |  | | | | |  |
| Model II | -0.027  (-0.044,-0.009) | **0.004** |  | | | | |  |
| Model III | -0.029  (-0.048,-0.011) | **0.002** |  | | | | |  |
| Model IV | -0.027  (-0.046,-0.009) | **0.004** |  | | | | |  |
| Model V | -0.017  (-0.035,0.001) | 0.06 |  | | | | |  |
| MDS = Mediterranean Diet Score; DASH = Dietary Approaches to Stop Hypertension.  Stepwise multiple linear regression values are expressed as unstandardised *β*-coefficients (95% confidence intervals), *P* values, R^2^ values and t values. Univariate linear regression models adjusted for age (continuous), sex (male/female) and smoking (yes/no)(model I), model I and Alcohol Use Disorders Identification Test Consumption score (continuous)(model II), model II and energy kcal/day (model III), model III and psychological morbidity (yes/no) (model IV), and model IV and body mass index (continuous) (model V). | | | | | | | |  |
