# Supplementary material for: Associations between diet quality indices and psoriasis severity: results from the Asking People with Psoriasis about Lifestyle and Eating (APPLE) cross-sectional study
Source: Br J Nutr. 2025 Feb 20;133(4):546–57. doi: 10.1017/S0007114525000340 (PMC12011542; doi:10.1017/S0007114525000340)

Version 1.0 16-Jan-23 324671

The APPLE Study

Asking **P**eople with **P**soriasis about **L**ifestyle and **E**ating.

We are looking for volunteers to help us understand if dietary & lifestyle patterns can affect psoriasis!

The study involves the completion of :

- an online multiple-choice survey →
- an online diet diary

You will receive:

- a personalised nutrition report
- an invite to "A Guide to Nutrition & Psoriasis" webinar

For more information:

dietandpsoriasisproject@kcl.ac.uk

To enroll:

https://dietandpsoriasisproject-pics.com

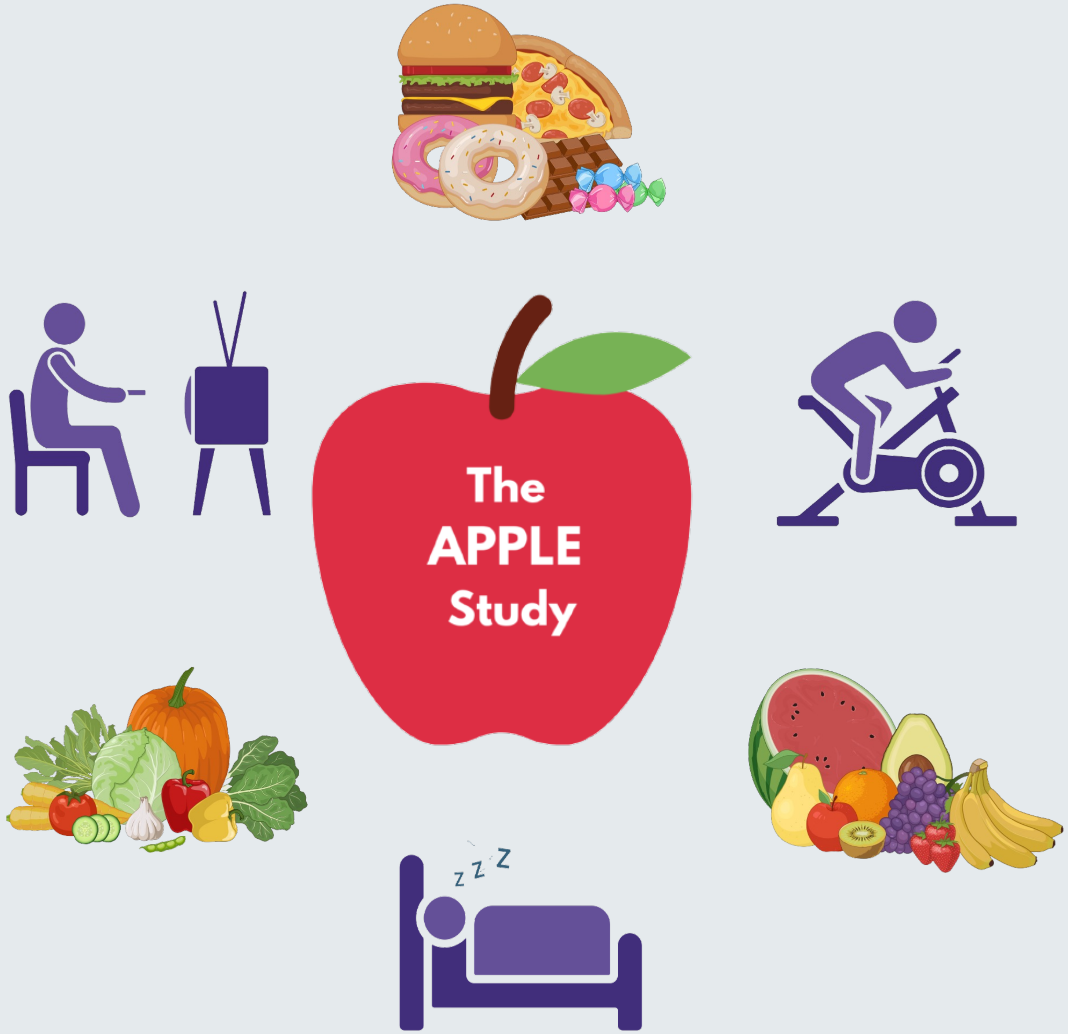

Supplement: Zanesco et al. supplementary material 3 — Zanesco et al. supplementary material [file S0007114525000340sup003.pdf]
