# Supplementary material for: Associations between diet quality indices and psoriasis severity: results from the Asking People with Psoriasis about Lifestyle and Eating (APPLE) cross-sectional study
Source: Br J Nutr. 2025 Feb 20;133(4):546–57. doi: 10.1017/S0007114525000340 (PMC12011542; doi:10.1017/S0007114525000340)
Supplement: Zanesco et al. supplementary material 4 — Zanesco et al. supplementary material [file S0007114525000340sup004.docx]

| **Supplementary Information 4.** European Prospective Investigation into Cancer Food Frequency Questionnaire items (n=147). |
| --- |
| **DAIRY PRODUCTS AND FATS** |
| Single or sour cream (tablespoon) |
| Double or clotted cream (tablespoon) |
| Low fat yoghurt, fromage frais (small pot) |
| Full fat or Greek yoghurt (small pot) |
| Dairy desserts (small pot) e.g. chocolate mousse, cream caramels |
| Cheese, e.g. cheddar, brie, edam (matchbox size) |
| Low fat cheese e.g. reduced fat cheddar (matchbox size) |
| Cottage cheese, low fat soft cheese (2 tablespoons) |
| Eggs as boiled, fried, scrambled, etc. (one) |
| Quiche (slice) |
| Low calorie, low fat salad cream (tablespoon) |
| Full fat salad cream, mayonnaise (tablespoon) |
| French dressing (tablespoon) |
| Other salad dressing (tablespoon) |
| Butter (teaspoon) |
| Reduced fat butter (teaspoon) |
| Block margarine, e.g. Stork, Krona (teaspoon) |
| Polyunsaturated margarine, e.g. Flora sunflower (teaspoon) |
| Olive oil spread (teaspoon) |
| Other soft margarine, dairy spreads, e.g. Blue Band, Clover (teaspoon) |
| Low fat spread, e.g. outline, Gold (teaspoon) |
| Very low fat spread (teaspoon) e.g. Diet Flora |
| Cholesterol lowering fat spreads e.g. Benecol (teaspoon) |

| **FRUIT** |
| --- |
| Apples (1 fruit) |
| Pears (1 fruit) |
| Oranges, satsumas, mandarins |
| Grapefruit (half) |
| Bananas (1 fruit) |
| Grapes (handful) |
| Melon (1 slice) |
| *Peaches, plums, apricots (1 fruit) |
| *Strawberries, raspberries, other berries, kiwi fruit (one fruit or handful) |
| Tinned fruit (handful) |
| Dried fruit, e.g. raisins, prunes (heaped tablespoon) |

| **VEGETABLES** |
| --- |
| Carrots |
| Spinach |
| Broccoli, spring green, kale |
| Brussels sprouts |
| Cabbage |
| Peas |
| Green beans, broad beans, runner beans |
| Baked beans |
| Marrow, courgettes |
| Cauliflower |
| Parsnips, turnips, swedes |
| Leeks |
| Onions |
| Garlic (clove) |
| Mushrooms |
| Sweet peppers |
| Beansprouts |
| Green salad, lettuce, cucumber, celery |
| Watercress |
| Tomatoes |
| Sweetcorn |
| Beetroot |
| Coleslaw |
| Avocado |
| Pulses e.g. lentils, beans, peas |
| Meat substitutes e.g. tofu, soyameat, textured vegetable protein, vegeburger |

| **MEAT AND FISH** |
| --- |
| Beef: roast, steak, mince, stew or casserole |
| Beefburgers |
| Pork: roast, chops or stew |
| Lamb: roast, chops or stew |
| Chicken or other poultry e.g. turkey |
| Bacon or gammon |
| Ham, cured meats & chorizo |
| Corned beef, Spam, luncheon meats |
| Sausages |
| Savoury pies, e.g. meat pie, pork pie, pastries, steak & kidney pie, sausage rolls |
| Liver, liver pate, liver sausage |
| Fried fish in batter, as fish and chips |
| Fish fingers, fish cakes & breaded fish |
| Other white fish, fresh or frozen, e.g. cod, plaice, sole, haddock, halibut |
| Oily fish, fresh or canned, e.g. tuna, mackerel, kippers, salmon, sardines, herring |
| Shellfish, e.g. crab, prawns, mussels |
| Fish roe, taramasalata |

| **BREAD AND SAVOURY BISCUITS** |
| --- |
| White bread/rolls |
| Brown bread/rolls |
| Wholemeal & granary bread/rolls |
| Cream crackers, savoury biscuits |
| Crispbread, e.g. Ryvita |
| Naan, poppadoms, flour tortillas |

| **SOUPS, SAUCES AND SPREADS** |
| --- |
| Vegetable soups (bowl) |
| Meat soups (bowl) (to include meat and vegetable soups) |
| Sauces, e.g. white sauce, cheese sauce, gravy (tablespoon) |
| Tomato ketchup (tablespoon) |
| Pickles, chutney (tablespoon) |
| Marmite, Bovril (teaspoon) |
| Jam, marmalade, honey (teaspoon) |
| Peanut butter (teaspoon) |

| **SWEETS AND SNACKS** |
| --- |
| Cakes e.g. fruit, sponge, home baked |
| Cakes e.g. fruit, sponge, ready made |
| Buns, pastries e.g. scones, flapjacks, croissants, doughnuts, home baked |
| Buns, pastries e.g. scones, flapjacks, croissants, doughnuts, ready made |
| Fruit pies, tarts, crumbles, home baked |
| Fruit pies, tarts, crumbles, ready made |
| Sponge puddings, home baked |
| Sponge puddings, ready made |
| Milk puddings e.g. rice, custard, trifle |
| Ice cream, choc ices |
| Sweet biscuits, chocolate, e.g. digestive (one) |
| Sweet biscuits, plain, e.g. Nice, ginger (one) |
| Reduced fat biscuits e.g. Go Ahead, Highlights (one small packet or one small bar/biscuit) |
| Cereal bars (one) |
| White or milk chocolates, single or squares (one) |
| Dark chocolates, single or squares (one) |
| Chocolate snacks bars e.g. Mars, Crunchie (one) |
| Sweets, toffees, mints (small packet) |
| Sugar added to tea, coffee, cereal (teaspoon) |
| Crips or other packet snacks, e.g. Wotsits (one packet) |
| Salted nuts, e.g. peanuts, cashews (handful) |
| Unsalted nuts, e.g. brazil, walnuts (handful) |
| Seed e.g. Sunflower, pumpkin (tablespoon) |

| **CEREAL** |
| --- |
| Porridge, Readybreak, oats |
| Breakfast cereal e.g. Cornflakes, Rice Krispies |
| Sugar topped cereals e.g. Frosties |
| Muesli |
| High Fibre cereals e.g. Branflakes, All Bran, Fruit and Fibre |

| **POTATOES, RICE AND PASTA** |
| --- |
| Boiled, mashed, instant or one jacket potato |
| Chips, roast potatoes |
| Potato salad |
| White rice |
| Brown rice |
| White or green pasta, e.g. spaghetti, macaroni, noodles |
| Wholemeal pasta |
| Lasagne, moussaka |
| Pizza (one slice) |

| **DRINKS** |
| --- |
| Tea (cup) |
| Green tea (cup) |
| Fruit tea (cup) |
| Coffee, instant or ground (cup) |
| Coffee, decaffeinated (cup) |
| Coffee whitener, e.g. Coffee-mate (teaspoon) |
| Cocoa, hot chocolate (cup) |
| Low fat hot chocolate (cup) |
| Horlicks, Ovaltine (cup) |
| White wine (small glass) |
| Red wine (small glass) |
| Beer, lager or cider (half pint) |
| Port, sherry, vermouth, liqueurs (pub measure) |
| Spirits, e.g. gin, brandy, whisky, vodka (pub measure) |
| Low calorie or diet fizzy soft drinks (cup) |
| Fizzy soft drinks, e.g. Coca Cola, lemonade (cup) |
| Pure fruit juice (100%) e.g. orange, apple juice (cup) |
| Fruit squash or cordial (cup) |
| Smoothies (cup) |
