# Supplementary material for: Associations between diet quality indices and psoriasis severity: results from the Asking People with Psoriasis about Lifestyle and Eating (APPLE) cross-sectional study
Source: Br J Nutr. 2025 Feb 20;133(4):546–57. doi: 10.1017/S0007114525000340 (PMC12011542; doi:10.1017/S0007114525000340)
Supplement: Zanesco et al. supplementary material 5 — Zanesco et al. supplementary material [file S0007114525000340sup005.docx]

| **Supplementary Information 5.** Components of the diet quality indices.  The Mediterranean Diet Score (MDS) components. | |
| --- | --- |
| *^+^Positively scored* | |
| **Vegetables** | Carrots, spinach, broccoli or spring greens or kale, brussels sprouts, cabbage, marrow or courgettes, cauliflower, parsnips or turnips or swedes, leeks, onions, garlic, mushrooms, sweet peppers, green salad or lettuce or cucumber or celery, watercress, tomatoes, sweetcorn, beetroot. |
| **Fruits & nuts** | Apples, pears, oranges or satsumas or mandarins, grapefruit, bananas, grapes, melon, peaches or plums or apricots, strawberries or raspberries or kiwi fruit, tinned fruit, dry fruit, avocado, salted nuts, unsalted nuts, seeds, peanut butter. |
| **Wholegrain cereals** | Brown bread rolls, wholemeal and granary bread and rolls, porridge, muesli, high-fibre cereal, brown rice, wholemeal pasta. |
| **Legumes** | Peas, green beans or broad beans or runner beans or baked beans, beansprouts, dried lentils or beans or peas. |
| **Fish or seafood** | Fried fish in batter, fish fingers or fish cakes or breaded fish, other white fish fresh or frozen, oily fish fresh or canned, shellfish, fish roe. |
| *^-^ Negatively scored* | |
| **Dairy products** | Single or sour cream, double or clotted cream, low fat yogurt, full fat or Greek yogurt, cheese, low fat cheese, cottage cheese. |
| **Meat and poultry products** | Beef roast or steak or mince, or stew or casserole, beef burgers, pork roast or chops or stew, lamb roast or chops or stew, chicken or other poultry, bacon or gammon, ham or cured meats or chorizo, corned beef or spam or luncheon meats, sausages, liver or liver pâté or liver sausage. |
| *Ratio-based score* | |
| **Alcohol** | 1 point = ≤16g; 0 points = >16g |
| ^†^**MS ratio** | 1 point = > 1; 0 points = < 1 |
| MS = Monounsaturated-to-saturated fat ratio  ^+^Positively scored components are allocated 1 point for intakes ≥ sex-specific median and 0 points for intakes < sex-specific median.  ^-^ Negatively scored components are allocated 1 point for intakes < sex-specific median and 0 points for intakes ≥ sex-specific median.  ^†^ Monounsaturated-to-saturated fat ratio; is calculated by diving the intake of monounsaturated fats by the intake of saturated fats. | |

| The Dietary Approaches to Stop Hypertension (DASH) score components. | |
| --- | --- |
| *^+^Positively scored* | |
| **Fruits** | Apples, pears, oranges or satsumas or mandarins, grapefruit, bananas, grapes, melon, peaches or plums or apricots, strawberries or raspberries or kiwi fruit, tinned fruit, dry fruit, avocado. |
| **Vegetables** | Carrots, spinach, broccoli or spring greens or kale, brussels sprouts, cabbage, marrow or courgettes, cauliflower, parsnips or turnips or swedes, leeks, onions, garlic, mushrooms, sweet peppers, green salad or lettuce or cucumber or celery, watercress, tomatoes, sweetcorn, beetroot. |
| **Nuts and Legumes** | Salted nuts, unsalted nuts, seeds, peanut butter, peas, green beans or broad beans or runner beans or baked beans, beansprouts, dried lentils or beans or peas. |
| **Wholegrain cereals** | Brown bread rolls, wholemeal and granary bread and rolls, porridge, muesli, high-fibre cereal, brown rice, wholemeal pasta. |
| **Low fat dairy products** | Low fat yogurt, low fat cheese, low fat cottage cheese. |
| *^-^ Negatively scored* | |
| **Sodium (g/day)** |  |
| **Red and processed meat products** | Beef roast or steak or mince, or stew or casserole, beef burgers, pork roast or chops or stew, lamb roast or chops or stew, bacon or gammon, ham or cured meats or chorizo, corned beef or spam or luncheon meats, sausages, liver or liver pâté or liver sausage. |
| **Sweetened beverages** | Low calorie or diet fizzy drinks, fizzy soft drinks, fruit squash or cordial. |
| ^+^Positively scored components are ranked in quintiles of intake; 1 point for the lowest quintile and up to 5 points for the highest quintile.  ^-^ Negatively scored components are ranked in quintiles of intake; 5 points for the lowest quintile and 1 point for the highest quintile. | |

| The Plant-based Diet Index (PDI) components. | | | | | | |
| --- | --- | --- | --- | --- | --- | --- |
| Components | FFQ variables | Point allocation | | | | |
|  |  | oPDI | hPDI | | uPDI | |
| *Healthy plant food group* | | | | | | |
| **Whole grain cereals** | Brown bread rolls, wholemeal and granary bread and rolls, porridge, muesli, high-fibre cereal, brown rice, wholemeal pasta. | + | + | - | | |
| **Fruits** | Apples, pears, oranges or satsumas or mandarins, grapefruit, bananas, grapes, melon, peaches or plums or apricots, strawberries or raspberries or kiwi fruit, tinned fruit, dry fruit, avocado. | + | + | - | | |
| **Vegetables** | Carrots, spinach, broccoli or spring greens or kale, brussels sprouts, cabbage, marrow or courgettes, cauliflower, parsnips or turnips or swedes, leeks, onions, garlic, mushrooms, sweet peppers, green salad or lettuce or cucumber or celery, watercress, tomatoes, sweetcorn, beetroot. | + | + | - | | |
| **Nuts** | Salted nuts, unsalted nuts, seeds, peanut butter. | + | + | - | | |
| **Legumes** | Peas, green beans or broad beans or runner beans or baked beans, beansprouts, dried lentils or beans or peas. | + | + | - | | |
| **Tea and Coffee** | Tea, green tea, fruit tea, coffee, decaffeinated coffee. | + | + | - | | |
| *Less healthy plant food group* | | | | | | |
| **Fruit juices** | Pure fruit juice, fruit squash or cordial. | + | - | | | + |
| **Refined grains** | White bread and rolls, cream crackers or savoury biscuits, crispbread, naan or poppadoms or flour tortillas, breakfast cereal, sugar-topped breakfast cereal, white rice, white or green pasta, reduced fat biscuits, cereal bars. | + | - | | | + |
| **Potatoes** | Boiled or mashed or instant or jacket potato, chips or roast potatoes, potato salad, crisps or packet snacks . | + | - | | | + |
| **Sugar-sweetened beverages** | Low calorie or diet fizzy soft drinks, fizzy soft drinks. | + | - | | | + |
| **Sweets and desserts** | Sweet biscuits (chocolate & plain), cakes (home-baked and ready-made), buns and pastries (home-baked and ready-made) fruits pies or tarts or crumbles (home-baked and ready-made) sponge puddings (home-baked and ready-made), white or milk chocolates, dark chocolates, chocolate snacks, chutney, jams or marmalade or honey. | + | - | | | + |
| *Animal food group* | | | | | | |
| **Animal fats** | Butter, reduced fat butter | - | - | | | - |
| **Dairy** | Single or sour cream, double or clotted cream, low fat yogurt, full fat or Greek yogurt, cheese, low fat cheese, cottage cheese, ice-cream or choc ices. | - | - | | | - |
| **Eggs** | Eggs as boiled, fried, or scrambled. | - | - | | | - |
| **Fish or seafood** | Fried fish in batter, fish fingers or fish cakes or breaded fish, other white fish fresh or frozen, oily fish fresh or canned, shellfish, fish roe. | - | - | | | - |
| **Meat** | Beef roast or steak or mince, or stew or casserole, beef burgers, pork roast or chops or stew, lamb roast or chops or stew, chicken or other poultry, bacon or gammon, ham or cured meats or chorizo, corned beef or spam or luncheon meats, sausages, liver or liver pâté or liver sausage. | - | - | | | - |
| **Miscellaneous animal-based foods** | Savoury pies, lasagne, pizza, quiche, low-fat salad cream, full fat salad cream or mayonnaise, meat soups. | - | - | | | - |
| FFQ = Food Frequency Questionnaire; oPDI = original Plant-based Diet Index; hPDI = healthy Plant-based Diet Index; uPDI = unhealthy Plant-based Diet Index.  Positively scored components (+) are ranked in quintiles of intake; 1 point for the lowest quintile and 5 points for the highest quintile.  Negatively scored components (-) are ranked in quintiles of intake; 5 points for the lowest quintile and 1 point for the highest quintile | | | | | |  |
