# Supplementary material for: Associations between diet quality indices and psoriasis severity: results from the Asking People with Psoriasis about Lifestyle and Eating (APPLE) cross-sectional study
Source: Br J Nutr. 2025 Feb 20;133(4):546–57. doi: 10.1017/S0007114525000340 (PMC12011542; doi:10.1017/S0007114525000340)
Supplement: Zanesco et al. supplementary material 6 — Zanesco et al. supplementary material [file S0007114525000340sup006.docx]

| **Supplementary Information 6.** the mean (standard deviation) for sa-SPI and MDS tertiles, and DASH and PDI quintiles. | | | | | |
| --- | --- | --- | --- | --- | --- |
|  |  | Category | *n* (%) | Mean (SD) DQI score | Mean (SD) sa-SPI score |
| **sa-SPI** | T_1_ | Lower severity | 83 (32) |  | 6.31 (4.87) |
|  | T_2_ | Increasing severity | 85 (33) |  | 16.71 (2.36) |
|  | T_3_ | Higher severity | 88 (35) |  | 27.21 (4.98) |
| **MDS** | T_1_ | Low adherence | 74 (29) | 2.69 (0.75) | 18.42 (10.47) |
|  | T_2_ | Modest adherence | 99 (38) | 4.58 (0.42) | 17.89 (9.02) |
|  | T_3_ | High adherence | 84 (33) | 6.54 (0.87) | 14.54 (8.90) |
| **DASH** | Q_1_ | Very low adherence | 49 (19) | 15.95 (2.41) | 21.33 (9.61) |
|  | Q_2_ | Low adherence | 54 (21) | 20.79 (0.97) | 18.37 (10.00) |
|  | Q_3_ | Modest adherence | 49 (19) | 23.79 (0.74) | 16.04 (7.86) |
|  | Q_4_ | High adherence | 49 (19) | 26.61 (0.83) | 14.83 (8.66) |
|  | Q_5_ | Very high adherence | 56 (22) | 31.50 (2.55) | 14.37 (9.82) |
| **oPDI** | Q_1_ | Very low adherence | 53 (21) | 41.23 (3.25) | 19.71 (11.03) |
|  | Q_2_ | Low adherence | 42 (16) | 47.15 (0.98) | 18.67 (8.09) |
|  | Q_3_ | Modest adherence | 60 (23) | 51.05 (1.22) | 15.99 (9.92) |
|  | Q_4_ | High adherence | 51 (20) | 55.36 (1.24) | 16.07 (8.53) |
|  | Q_5_ | Very high adherence | 50 (20) | 61.62 (3.16) | 14.49 (8.91) |
| **hDPI** | Q_1_ | Very low adherence | 52 (20) | 39.69 (4.01) | 18.26 (9.66) |
|  | Q_2_ | Low adherence | 48 (19) | 47.33 (1.43) | 19.24 (9.25) |
|  | Q_3_ | Modest adherence | 53 (21) | 52.13 (1.37) | 18.20 (9.70) |
|  | Q_4_ | High adherence | 49 (19) | 56.99 (1.48) | 15.78 (9.50) |
|  | Q_5_ | Very high adherence | 54 (21) | 64.74 (4.04) | 13.63 (8.85) |
| **uPDI** | Q_1_ | Very low adherence | 48 (18) | 39.62 (3.64) | 16.53 (10.00) |
|  | Q_2_ | Low adherence | 49 (20) | 46.72 (1.39) | 14.14 (8.76) |
|  | Q_3_ | Modest adherence | 54 (21) | 51.34 (1.28) | 17.72 (9.96) |
|  | Q_4_ | High adherence | 51 (20) | 55.89 (1.37) | 17.34 (9.93) |
|  | Q_5_ | Very high adherence | 54 (21) | 63.08 (3.71) | 18.53 (8.81) |
| DQI = Diet Quality Index; sa-SPI = self-assessed Simplified Psoriasis Index; MDS = Mediterranean Diet Score; DASH = Dietary Approaches to Stop Hypertension; oPDI = original Plant-based Diet Index; hPDI = healthy Plant-based Diet Index; uPDI = unhealthy Plant-based Diet Index. n=1 excluded from rank transformation for sa-SPI (59 points), n=1 excluded from rank transformation for oPDI (73 points), n=1 excluded from rank transformation for hPDI (76 points), and n=1 excluded from rank transformation for uPDI (73 points). | | | | | |
