# Supplementary material for: Associations between diet quality indices and psoriasis severity: results from the Asking People with Psoriasis about Lifestyle and Eating (APPLE) cross-sectional study
Source: Br J Nutr. 2025 Feb 20;133(4):546–57. doi: 10.1017/S0007114525000340 (PMC12011542; doi:10.1017/S0007114525000340)
Supplement: Zanesco et al. supplementary material 7 — Zanesco et al. supplementary material [file S0007114525000340sup007.docx]

| **Supplementary Information 7.** Demographic comparisons between participants with complete and incomplete survey responses. | | | |
| --- | --- | --- | --- |
|  | **Complete**  **(n=269)** | **Incomplete**  **(n=84)** | |
| Age, years (median, IQR) | 40 (20) | | 40 (18) |
| BMI (median, IQR) | 25 (8) | | 28 (10) |
| Psoriasis severity (median, IQR) | 15 (12) | | 13 (18) |
| **Gender (N, %)** | | | |
| Male | 49 (18.2) | | 15 (17.9) |
| Female | 219 (81.4) | | 69 (82.1) |
| Non-binary | 1 (0.4) | | 0 (0.0) |
| **Body Mass Index (BMI) classification (N, %)** | | | |
| Underweight | 7 (2.6) | | 0 (0.0) |
| Normal weight | 122 (45.9) | | 28 (34.1) |
| Overweight | 70 (26.3) | | 21 (25.6) |
| Obese | 67 (25.2) | | 33 (40.3) |
| **Ethnicity (N, %)** | | | |
| White - British | 228 (84.5) | | 71 (84.5) |
| White (Other) | 16 (6.6) | | 7 (8.3) |
| Mixed | 10 (3.7) | | 1 (1.2) |
| South Asian | 9 (3.3) | | 3 (3.6) |
| Asian (Other) | 3 (1.1) | | 0 (0.0) |
| East Asian | 1 (0.4) | | 2 (2.4) |
| Black-Caribbean | 1 (0.4) | | 0 (0.0) |
| **Smoking status (N,%)** | | | |
| Non-smoking | 221 (82.2) | | 79 (89.4) |
| Actively smoking | 47 (17.4) | | 7 (10.6) |
| Preferred not to say | 1 (0.4) | | 0 (0.0) |
| **Alcohol overconsumption (N,%)** | | | |
| Low risk of dependency | 160 (59.5) | | 40 (54.0) |
| Increasing risk of dependency | 86 (32.0) | | 21 (28.4) |
| Higher risk of dependency | 20 (7.4) | | 13 (17.6) |
| Possible dependence | 3 (1.1) | | 0 (0.0) |
| **Family history of psoriasis (N, %)** | | | |
| Yes | 143 (53.2) | | 38 (45.2) |
| No | 126 (46.8) | | 46 (54.8) |
| **Morbidity (N, %)** | | | |
| *Psoriatic Arthritis* |  | |  |
| Yes | 57 (21.2) | | 20 (23.8) |
| No | 212 (78.8) | | 64 (76.2) |
| *Cardiometabolic* |  | |  |
| Yes | 50 (18.6) | | 12 (14.3) |
| No | 219 (81.4) | | 72 (85.7) |
| *Psychological* |  | |  |
| Yes | 121 (45.0) | | 22 (26.2) |
| No | 148 (55.0) | | 62 (73.8) |
| *Gastrointestinal* |  | |  |
| Yes | 57 (21.2) | | 15 (17.9) |
| No | 212 (78.8) | | 79 (82.1) |
| **Psoriasis severity (N, %)** | | | |
| Mild | 64 (3.8 | | 18 (34.0) |
| Moderate | 119 (44.2) | | 19 (35.8) |
| Severe | 86 (32.0) | | 16 (30.2) |
| Underweight BMI ≤17.99 kg/m^2^; normal weight BMI >18.00 kg/m^2^ and ≤24.99 kg/m^2^; overweight BMI >25.00 kg/m^2^ and ≤29.99 kg/m^2^; obese BMI >30.00 kg/m^2^.  Low risk of dependency 0-4 points; increasing risk of dependency 5-7 points; higher risk of dependency 8-10 points; possible dependence 11-12 points. Cardiometabolic morbidity includes one or more diagnoses of; heart disease, liver disease, stroke, type II diabetes, high blood pressure, high cholesterol or metabolic syndrome. Psychological morbidity includes a diagnosis of depression or anxiety. Gastrointestinal morbidity includes a diagnosis of irritable bowel syndrome, inflammatory bowel disease, or Celiac disease.  Psoriasis severity determined using the self-assessed Simplified Psoriasis Index.  Mild psoriasis 0-9.99 points; moderate psoriasis 10-19.99 points; severe psoriasis >20 points.  For participants with complete responses: n=3 missing values for BMI.  For participants with complete responses: n=2 missing values for BMI. n=18 missing values for smoking status.  n=10 missing values for alcohol overconsumption. n=31 missing values for psoriasis severity. | | | |
