# Supplementary material for: Associations between diet quality indices and psoriasis severity: results from the Asking People with Psoriasis about Lifestyle and Eating (APPLE) cross-sectional study
Source: Br J Nutr. 2025 Feb 20;133(4):546–57. doi: 10.1017/S0007114525000340 (PMC12011542; doi:10.1017/S0007114525000340)
Supplement: Zanesco et al. supplementary material 8 — Zanesco et al. supplementary material [file S0007114525000340sup008.docx]

| **Supplementary Information 8**: Diet quality indices and the adjusted OR (95% CI) for psoriasis severity (Model I-III). | | | | | | | | | | | |
| --- | --- | --- | --- | --- | --- | --- | --- | --- | --- | --- | --- |
|  |  |  | Model I | | | Model II | | | Model lII | | |
|  |  | cases/n | OR | 95% CI | *P* | OR | 95% CI | *P* | OR | 95% CI | *P* |
| **MDS**  **tertiles** | **Increased severity (T_2_) vs low severity (T_1_)** |  |  |  |  |  |  |  |  |  |  |
|  | T_1_ low adherence | 22/85 | 0.96 | 0.438 – 2.096 | 0.915 | 0.97 | 0.439 – 2.161 | 0.949 | 1.03 | 0.458 – 2.294 | 0.952 |
|  | T_2_ modest adherence | 33/85 | 1.31 | 0.638 – 2.699 | 0.461 | 1.33 | 0.639– 2.774 | 0.445 | 1.37 | 0.656 – 2.878 | 0.399 |
|  | T_3_ high adherence | 30/85 | Ref. |  |  |  |  |  |  |  |  |
|  | **High severity (T_3_) vs low severity (T_1_)** |  |  |  |  |  |  |  |  |  |  |
|  | T_1_ low adherence | 28/88 | 1.91 | 0.861 – 4.222 | 0.112 | 1.98 | 0.882 – 4.459 | 0.098 | 2.07 | 0.912 – 4.698 | 0.082 |
|  | T_2_ modest adherence | 39/88 | 2.28 | 1.081 – 4.811 | **0.030** | 2.36 | 1.106 – 5.052 | **0.03** | 2.43 | 1.130 – 5.214 | **0.02** |
|  | T_3_ high adherence | 21/88 | Ref. |  |  |  |  |  |  |  |  |
| **DASH**  **quintiles** | **Increased severity (T_2_) vs low severity (T_1_)** |  |  |  |  |  |  |  |  |  |  |
|  | Q_1_ Very low adherence | 15/85 | 2.04 | 0.703 – 5.921 | 0.189 | 2.06 | 0.707 – 5.970 | 0.186 | 2.07 | 0.710 – 6.007 | 0.183 |
|  | Q_2_ Low adherence | 13/85 | 0.91 | 0.350 – 2.388 | 0.855 | 0.92 | 0.350 – 2.391 | 0.856 | 0.94 | 0.357 – 2.453 | 0.894 |
|  | Q_3_ Modest adherence | 19/85 | 1.59 | 0.623 – 4.055 | 0.332 | 1.62 | 0.630 – 4.160 | 0.317 | 1.65 | 0.641 – 4.255 | 0.299 |
|  | Q_4_ High adherence | 21/85 | 1.64 | 0.661 – 4.045 | 0.288 | 1.65 | 0.665 – 4.084 | 0.281 | 1.68 | 0.674 – 4.166 | 0.266 |
|  | Q_5_ Very high adherence | 17/85 | Ref. |  |  |  |  |  |  |  |  |
|  | **High severity (T_3_) vs low severity (T_1_)** |  |  |  |  |  |  |  |  |  |  |
|  | Q_1_ Very low adherence | 24/88 | 4.09 | 1.455 – 11.494 | **0.008** | 4.10 | 1.457 – 11.539 | **0.008** | 4.10 | 1.458 – 11.547 | **0.008** |
|  | Q_2_ Low adherence | 23/88 | 1.87 | 0.757 – 4.604 | 0.175 | 1.87 | 0.758 – 4.606 | 0.175 | 1.89 | 0.764 – 4.661 | 0.169 |
|  | Q_3_ Modest adherence | 14/88 | 1.38 | 0.514 – 3.693 | 0.524 | 1.39 | 0.513 – 3.736 | 0.520 | 1.40 | 0.518 – 3.779 | 0.508 |
|  | Q_4_ High adherence | 11/88 | 0.96 | 0.351 – 2.595 | 0.928 | 0.96 | 0.352 – 2.603 | 0.931 | 0.97 | 0.354 – 2.629 | 0.944 |
|  | Q_5_ Very high adherence | 16/88 | Ref. |  |  |  |  |  |  |  |  |
| **oPDI**  **quintiles** | **Increased severity (T_2_) vs low severity (T_1_)** |  |  |  |  |  |  |  |  |  |  |
|  | Q_1_ Very low adherence | 17/85 | 1.19 | 0.445 – 3.162 | 0.733 | 1.19 | 0.445 – 3.162 | 0.733 | 1.42 | 0.498 – 4.034 | 0.514 |
|  | Q_2_ Low adherence | 12/85 | 0.84 | 0.305 – 2.317 | 0.736 | 0.84 | 0.304 – 2.332 | 0.741 | 0.94 | 0.331 – 2.665 | 0.906 |
|  | Q_3_ Modest adherence | 18/85 | 0.68 | 0.282 – 1.619 | 0.379 | 0.68 | 0.282 – 1.623 | 0.381 | 0.74 | 0.301 – 1.797 | 0.500 |
|  | Q_4_ High adherence | 16/85 | 0.86 | 0.336 – 2.212 | 0.758 | 0.87 | 0.335 – 2.230 | 0.763 | 0.89 | 0.344 – 2.317 | 0.816 |
|  | Q_5_ Very high adherence | 22/85 | Ref. |  |  |  |  |  |  |  |  |
|  | **High severity (T_3_) vs low severity (T_1_)** |  |  |  |  |  |  |  |  |  |  |
|  | Q_1_ Very low adherence | 22/87 | 4.09 | 1.391 – 12.019 | **0.01** | 4.09 | 1.391 – 12.020 | **0.01** | 5.10 | 1.628 – 15.954 | **0.005** |
|  | Q_2_ Low adherence | 18/87 | 3.06 | 1.035 – 9.025 | **0.04** | 3.07 | 1.034 – 9.107 | **0.04** | 3.52 | 1.155 – 10.700 | **0.03** |
|  | Q_3_ Modest adherence | 19/87 | 1.72 | 0.629 – 4.675 | 0.292 | 1.72 | 0.630 – 4.689 | 0.291 | 1.90 | 0.686 – 5.285 | 0.216 |
|  | Q_4_ High adherence | 19/87 | 2.70 | 0.944 – 7.715 | 0.064 | 2.71 | 0.943 – 7.788 | 0.064 | 2.81 | 0.972 – 8.115 | 0.057 |
|  | Q_5_ Very high adherence | 9/87 | Ref. |  |  |  |  |  |  |  |  |
| **hPDI**  **quintiles** | **Increased severity (T_2_) vs low severity (T_1_)** |  |  |  |  |  |  |  |  |  |  |
|  | Q_1_ Very low adherence | 21/85 | 2.09 | 0.746 – 5.828 | 0.161 | 2.06 | 0.732 – 5.770 | 0.171 | 2.00 | 0.678 – 5.924 | 0.209 |
|  | Q_2_ Low adherence | 14/85 | 1.06 | 0.386 – 2.882 | 0.918 | 1.05 | 0.385 – 2.877 | 0.921 | 1.04 | 0.375 – 2.875 | 0.942 |
|  | Q_3_ Modest adherence | 18/85 | 1.16 | 0.451 – 2.979 | 0.760 | 1.15 | 0.448 – 2.968 | 0.767 | 1.14 | 0.436 – 2.973 | 0.792 |
|  | Q_4_ High adherence | 11/85 | 0.49 | 0.191 – 1.276 | 0.145 | 0.48 | 0.186 – 1.262 | 0.138 | 0.48 | 0.185 – 1.258 | 0.136 |
|  | Q_5_ Very high adherence | 21/85 | Ref. |  |  |  |  |  |  |  |  |
|  | **High severity (T_3_) vs low severity (T_1_)** |  |  |  |  |  |  |  |  |  |  |
|  | Q_1_ Very low adherence | 20/88 | 4.31 | 1.429 – 12.984 | **0.009** | 4.28 | 1.416 – 12.951 | **0.01** | 4.69 | 1.468 – 14.967 | **0.009** |
|  | Q_2_ Low adherence | 20/88 | 3.14 | 1.110 – 8.850 | **0.03** | 3.13 | 1.109 – 8.842 | **0.03** | 3.28 | 1.145 – 9.415 | **0.03** |
|  | Q_3_ Modest adherence | 20/88 | 2.63 | 0.958 – 7.193 | 0.061 | 2.62 | 0.956 – 7.181 | 0.061 | 2.75 | 0.986 – 7.675 | **0.05** |
|  | Q_4_ High adherence | 16/88 | 1.30 | 0.490 – 3.396 | 0.606 | 1.28 | 0.482 – 3.393 | 0.621 | 1.30 | 0.490 – 3.471 | 0.595 |
|  | Q_5_ Very high adherence | 12/88 | Ref. |  |  |  |  |  |  |  |  |
| **uPDI**  **quintiles** | **Increased severity (T_2_) vs low severity (T_1_)** |  |  |  |  |  |  |  |  |  |  |
|  | Q_1_ Very low adherence | 19/85 | 0.86 | 0.299 – 2.468 | 0.777 | 0.86 | 0.299 – 2.469 | 0.777 | 0.81 | 0.277 – 2.351 | 0.695 |
|  | Q_2_ Low adherence | 13/85 | 0.33 | 0.120 – 0.929 | **0.04** | 0.33 | 0.120 – 0.930 | **0.04** | 0.32 | 0.116 – 0.907 | **0.03** |
|  | Q_3_ Modest adherence | 16/85 | 0.66 | 0.232 – 1.883 | 0.438 | 0.66 | 0.232 – 1.888 | 0.440 | 0.66 | 0.231 – 1.885 | 0.437 |
|  | Q_4_ High adherence | 17/85 | 0.53 | 0.196 – 1.455 | 0.220 | 0.54 | 0.196 – 1.459 | 0.222 | 0.54 | 0.197 – 1.465 | 0.225 |
|  | Q_5_ Very high adherence | 20/85 | Ref. |  |  |  |  |  |  |  |  |
|  | **High severity (T_3_) vs low severity (T_1_)** |  |  |  |  |  |  |  |  |  |  |
|  | Q_1_ Very low adherence | 15/87 | 0.51 | 0.172 – 1.497 | 0.219 | 0.51 | 0.172 – 1.497 | 0.219 | 0.49 | 0.164 – 1.457 | 0.199 |
|  | Q_2_ Low adherence | 11/87 | 0.23 | 0.079 – 0.642 | **0.005** | 0.23 | 0.079 – 0.642 | **0.005** | 0.22 | 0.077 – 0.632 | **0.005** |
|  | Q_3_ Modest adherence | 23/87 | 0.76 | 0.275 – 2.069 | 0.585 | 0.76 | 0.275 – 2.070 | 0.585 | 0.75 | 0.274 – 2.069 | 0.583 |
|  | Q_4_ High adherence | 16/87 | 0.42 | 0.152 – 1.133 | 0.086 | 0.42 | 0.152 – 1.133 | 0.086 | 0.42 | 0.152 – 1.136 | 0.087 |
|  | Q_5_ Very high adherence | 22/87 | Ref. |  |  |  |  |  |  |  |  |
| Results of the multinomial regression were expressed as Odds Ratios (OR) with 95% Confidence Intervals (CI).  MDS = Mediterranean Diet Score; DASH = Dietary Approaches to Stop Hypertension; oPDI = original Plant-based Diet Index; hPDI = healthy Plant-based Diet Index; uPDI = unhealthy Plant-based Diet Index.  The reference categories for the diet quality indices were “very high adherence” (DASH and PDIs) and “high adherence” (MDS).  Confounder adjustments:  Model I = age (continuous), sex (male/female) and smoking (yes/no).  Model II = Model I and Alcohol Use Disorders Identification Test Consumption score (continuous).  Model III = Model II and energy kcal/day (continuous).  sa-SPI tertiles: T_1_ (low severity) ≤ 7; T_2_ (increasing severity) 8 - 17; T_3_ (high severity) ≥ 18.  MDS tertiles: T_1_ (low adherence) ≤ 3; T_2_ (modest adherence) 4 - 5; T_3_ (high adherence) ≥ 6.  DASH quintiles = Q_1_ (very low adherence) ≤ 16; Q_2_ (low adherence) 17 - 20; Q_3_ (modest adherence) 21 - 24; Q_4_ (high adherence) 25 - 27; Q_5_ (very high adherence) ≥ 28.  oPDI quintiles = Q_1_ (very low adherence) ≤ 43; Q_2_ (low adherence) 44 - 47; Q_3_ (modest adherence) 48 - 51; Q_4_ (high adherence) 52 - 55; Q_5_ (very high adherence) ≥ 56.  hPDI quintiles = Q_1_ (very low adherence) ≤ 41; Q_2_ (low adherence) 42 - 47; Q_3_ (modest adherence) 48 - 52; Q_4_ (high adherence) 53 - 57; Q_5_ (very high adherence) ≥ 58.  uPDI quintiles = Q_1_ (very low adherence) ≤ 41; Q_2_ (low adherence) 42 - 48; Q_3_ (modest adherence) 49 - 51; Q_4_ (high adherence) 52 - 56; Q_5_ (very high adherence) ≥ 57. | | | | | | | | | | | |
