# Supplementary material for: Associations between diet quality indices and psoriasis severity: results from the Asking People with Psoriasis about Lifestyle and Eating (APPLE) cross-sectional study
Source: Br J Nutr. 2025 Feb 20;133(4):546–57. doi: 10.1017/S0007114525000340 (PMC12011542; doi:10.1017/S0007114525000340)
Supplement: Zanesco et al. supplementary material 9 — Zanesco et al. supplementary material [file S0007114525000340sup009.docx]

| **Supplementary Information 9.** mediation analysis examining the mediating effect of BMI on the DQI and psoriasis severity associations. | | | | | |
| --- | --- | --- | --- | --- | --- |
|  | Unstandardised - *β* | | 95% CI | *P* | Model tested |
| MDS → BMI | | -1.130 | -1.580 – -0.679 | **<0.001** | Exposure-Mediator |
| MDS + BMI → sa-SPI | |  |  |  |  |
| MDS | | -0.477 | -1.172 – 0.218 | 0.178 | Direct effect |
| BMI | | 0.504 | 0.320 – 0.688 | **<0.001** |  |
| MDS → sa-SPI | | -1.060 | -1.771 – 0.348 | **0.004** | Total effect |
| DASH → BMI | | -0.476 | -0.604 – -0.348 | **<0.001** | Exposure-Mediator |
| DASH + BMI → sa-SPI | |  |  |  |  |
| DASH | | -0.226 | -0.443 – -0.009 | **0.04** | Direct effect |
| BMI | | 0.457 | 0.265 – 0.650 | **<0.001** |  |
| DASH → sa-SPI | | -0.440 | -0.644 – -0.236 | **<0.001** | Total effect |
| oPDI → BMI | | -0.189 | -0.294 – -0.083 | **<0.001** | Exposure-Mediator |
| oPDI + BMI → sa-SPI | |  |  |  |  |
| oPDI | | -0.132 | -0.288 – 0.023 | 0.094 | Direct effect |
| BMI | | 0.512 | 0.333 – 0.691 | **<0.001** |  |
| oPDI → sa-SPI | | -0.229 | -0.389 – -0.069 | **0.005** | Total effect |
| hPDI → BMI | | -0.267 | -0.348 – 0.187 | **<0.001** | Exposure-Mediator |
| hPDI + BMI → sa-SPI | |  |  |  |  |
| hPDI | | -0.089 | -0.221 – 0.044 | 0.188 | Direct effect |
| BMI | | 0.494 | 0.304 – 0.683 | **<0.001** |  |
| hPDI → sa-SPI | | -0.215 | -0.343 – 0.087 | **0.001** | Total effect |
| uPDI → BMI | | 0.194 | 0.103 – 0.286 | **<0.001** | Exposure-Mediator |
| uPDI + BMI → sa-SPI | |  |  |  |  |
| uPDI | | 0.029 | -0.109 – 0.168 | 0.679 | Direct effect |
| BMI | | 0.531 | 0.348 – 0.713 | **<0.001** |  |
| uPDI → sa-SPI | | 0.136 | -0.005 – 0.276 | 0.06 | Total effect |
| BMI = Body Mass Index; DQI = Diet Quality Index; sa-SPI = self-assessed Simplified Psoriasis Index; MDS = Mediterranean Diet Score; DASH = Dietary Approaches to Stop Hypertension; oPDI = original Plant-based Diet Index; hPDI = healthy Plant-based Diet Index; uPDI = unhealthy Plant-based Diet Index.  A 3-step mediation analysis was conducted. Step 1 = linear regression with DQI (as the independent variable) and BMI (as the dependent variable) for the exposure-mediator effect. Step 2 = a multiple linear regression with DQI and BMI (as the independent variables) and sa-SPI (as the dependent variable) for the direct effect. Step 3 = linear regression with DQI (as the independent variable) and sa-SPI (as the dependent variable) for the total effect. Partial mediation = if all unstandardised - *β* coefficients for a given DQI are statistically significant and the unstandardised - *β* coefficient of the DQI in the direct effect model is closer to zero than that of the DQI in the total effect model. Full mediation = if the unstandardised - *β* coefficient for a given DQI is not statistically significant within the direct effect model, but the mediator is statistically significant. The effect of the MDS, oPDI, and hPDI on sa-SPI is fully mediated by BMI and disappears when the BMI is added to the regression model. BMI partially mediates the effect of the DASH on sa-SPI meaning that when BMI is added to the model, the effect of DASH is reduced but not nullified. | | | | | |
